# Immune cell infiltration and inflammatory landscape in primary brain tumours

**DOI:** 10.1186/s12967-024-05309-1

**Published:** 2024-05-30

**Authors:** Amalia Luce, Marianna Abate, Giosuè Scognamiglio, Marco Montella, Domenico Iervolino, Severo Campione, Annabella Di Mauro, Orlando Sepe, Vincenzo Gigantino, Madhura S. Tathode, Gerardo Ferrara, Roberto Monaco, Gianfranco De Dominicis, Gabriella Misso, Vittorio Gentile, Renato Franco, Silvia Zappavigna, Michele Caraglia

**Affiliations:** 1 Department of Precision Medicine, University of Campania “Luigi Vanvitelli”, Via L. De Crecchio, 7, 80138 Naples, Italy; 2 Laboratory of Precision and Molecular Oncology, Biogem Scarl, Institute of Genetic Research, 83031 Ariano Irpino, Italy; 3 Pathological Anatomy and Cytopathology Unit, Istituto Nazionale Tumori, IRCCS Fondazione G. Pascale, 80131 Naples, Italy; 4 Department of Mental and Physical Health and Preventive Medicine, Pathology Unit, University of Campania “Luigi Vanvitelli”, 80138 Naples, Italy; 5 Department of Advanced Technology, Pathology Unit, Cardarelli Hospital, 80131 Naples, Italy

**Keywords:** Meningioma, Glioblastoma, Astrocytoma, Inflammation, 5-Lypoxygenase, Immune cell infiltration

## Abstract

**Background:**

Primary malignant brain tumours are more than one-third of all brain tumours and despite the molecular investigation to identify cancer driver mutations, the current therapeutic options available are challenging due to high intratumour heterogeneity. In addition, an immunosuppressive and inflammatory tumour microenvironment strengthens cancer progression. Therefore, we defined an immune and inflammatory profiling of meningioma and glial tumours to elucidate the role of the immune infiltration in these cancer types.

**Methods:**

Using tissue microarrays of 158 brain tumour samples, we assessed CD3, CD4, CD8, CD20, CD138, Granzyme B (GzmB), 5-Lipoxygenase (5-LOX), Programmed Death-Ligand 1 (PD-L1), O-6-Methylguanine-DNA Methyltransferase (MGMT) and Transglutaminase 2 (TG2) expression by immunohistochemistry (IHC). IHC results were correlated using a Spearman correlation matrix. Transcript expression, correlation, and overall survival (OS) analyses were evaluated using public datasets available on GEPIA2 in Glioblastoma (GBM) and Lower Grade Glioma (LGG) cohorts.

**Results:**

Seven out of ten markers showed a significantly different IHC expression in at least one of the evaluated cohorts whereas CD3, CD4 and 5-LOX were differentially expressed between GBMs and astrocytomas. Correlation matrix analysis revealed that 5-LOX and GzmB expression were associated in both meningiomas and GBMs, whereas 5-LOX expression was significantly and positively correlated to TG2 in both meningioma and astrocytoma cohorts. These findings were confirmed with the correlation analysis of TCGA-GBM and LGG datasets. Profiling of mRNA levels indicated a significant increase in CD3 (CD3D, CD3E), and CD138 (SDC1) expression in GBM compared to control tissues. CD4 and 5-LOX (ALOX5) mRNA levels were significantly more expressed in tumour samples than in normal tissues in both GBM and LGG. In GBM cohort, GzmB (GZMB), SDC1 and MGMT gene expression predicted a poor overall survival (OS). Moreover, in LGG cohort, an increased expression of CD3 (CD3D, CD3E, CD3G), CD8 (CD8A), GZMB, CD20 (MS4A1), SDC1, PD-L1, ALOX5, and TG2 (TGM2) genes was associated with worse OS.

**Conclusions:**

Our data have revealed that there is a positive and significant correlation between the expression of 5-LOX and GzmB, both at RNA and protein level. Further evaluation is needed to understand the interplay of 5-LOX and immune infiltration in glioma progression.

**Supplementary Information:**

The online version contains supplementary material available at 10.1186/s12967-024-05309-1.

## Introduction

Glioblastoma multiforme (GBM), anaplastic astrocytoma, meningioma, and oligodendroglial tumors represent the most frequently diagnosed primary brain tumors (PBTs) [1, 2]. The most part of PBTs are aggressive and resistant neoplasms and, although the etiology of most PBTs has not yet been fully clarified, risk factors such as ionizing radiations and hereditary cancer syndromes seem to play key roles [3, 4].

PBTs represent 1.7% of all cancers and meningiomas are the most frequent (36.8% of all neoplasms) whereas gliomas are the most common malignant type, representing 75% of the malignant central nervous system (CNS) tumours of adults [4]. Despite different treatment options, including neurosurgery, radiotherapy and chemotherapy, the overall survival is still poor, accounting for an aggregate 5-year survival rate of 20% taking also in consideration the new treatment modalities that have had a limited impact on the survival of the patients until now [2, 5].

It was formerly believed that the blood-brain barrier (BBB) and blood-cerebrospinal fluid barrier (BCB) provided a defense mechanism that blocked both anti-cancer drugs and immune cells from penetrating the brain from the outside creating an immunological refuge [6, 7]. However, recent studies highlighted the connection between cancer-related inflammation and immunotherapy [8–13]. In fact, neoangiogenesis in PBTs affects the cerebral vasculature inducing an increased permeabilization of the BBB and correlates with aggressiveness and tumour stemness in the CNS [14–16]. Neurodegenerative and inflammatory (immune-mediated and infectious) diseases at early stages can induce a dysfunction of BBB with cerebral edema and cellular infiltration into the brain [17]. It is presently commonly believed that the resistance of PBTs to immunological host defenses is likely due to still not identified resistance mechanisms of both PBT cells and microenvironment cell components that confer an intrinsically resistant phenotype to PBTs themselves. In fact, it was recently reported that ionizing radiation enhances the expression of immune-suppressive markers on GBM cells and induce in some tumor microenvironment (TME) cells, such as macrophages, the upregulation of programmed death-ligand 1 (PD-L1) in response to extracellular vesicles (EVs) released by GBM cells, and the increase of CD206^+^ macrophages with pro-oncogenic properties [18]. Moreover, it is emerging that both tumour microglia and myeloid cell derivatives can have an important role in forming a true immunological barrier against immunotherapy [19].

In line with these observations, pathways commonly activated in various human PBTs and promoting invasiveness and the expression of pro-inflammatory molecules [20] involved in the regulation of TME infiltrates are those mediated by 5-Lipoxygenases (5-LOX) [21, 22], COX-2 and cytokines [23, 24] with oncogenic properties [18]. Interestingly, a multifunctional enzyme, the transglutaminase 2 (TG2) might have a role in the inflammatory processes, through NF-κB upregulation and, mesenchymal transition promotion in GBM [25, 26]. In this context, previous research showed an increased CD4^+^ and CD8^+^ T cell and natural killer (NK) cell infiltration in the TME of lower grade gliomas (LGGs), and conversely a sparse infiltration in immunosuppressed high-grade gliomas (HGGs) [27–29]. Hosseinalizadeh et al. reported that the expression of indoleamine 2,3-dioxygenase in GBM cells induced an activation of immunosuppressive and malignant regulatory T cells and a decreased expression of cytotoxic T lymphocytes (CTLs) [30]. Binding PD-1 receptor expressed on CD8^+^ T cells, programmed death-ligand 1 (PD-L1) can suppress NK and T cell functions inducing tumour escape from the immune system surveillance [31]. Although PD-L1 expression is inversely correlated with tumor-infiltrating CD8^+^ T-cells in GBM patients [32, 33], the role of PD-L1 expression as prognostic factor in PBTs is not fully clarified. Xue et al. reported that the positive PD-L1 protein expression varies in different studies ranging from 6.1% to 100% [34]. In meningioma, PD-L1 has been associated with radiation therapy failure response and identified as a marker of recurrence prediction [35, 36]. Moreover, the intratumoral density of proliferating CD8^+^ T cells and higher CD8^+^/CD4^+^ ratios are considered independent predictive factors for improved overall survival (OS) in GBM patients [37]. Mu et al. demonstrated that CD4^+^ was the predominant T cell population in primary GBM, highly expressed also in bevacizumab-resistant recurrent tumors, and together with perivascular Foxp3^+^ T cells correlated with angiogenesis in glioma [38]. CD20 is a B cell-specific marker, generally low expressed in the tumour infiltrating and tumour areas, but highly expressed in the vascular areas in GBM [39, 40]. CD138 or syndecan-1 (SDC1) is one of four members of the syndecan family. CD138 expression differs among cancer types and tumor aggressiveness and clinical outcomes are highly correlated with its differential expression in stromal compartments and carcinoma cells [41–43]. Particularly, SDC1 expression in human gliomas corresponds with advanced tumor progression and poor prognosis [44]. Granzyme B (GzmB) is a serine protease associated with various diseases like viral infections, autoimmunity, transplant rejection, and anti-tumor immunity [45]. Recent studies reveal a significant overexpression of GZMB in high-grade glioma patients compared to low-grade glioma (LGG) cases [46]. GZMB serves also as an independent prognostic biomarker for GBM, showing positive correlations with immunoinhibitors, immunostimulants, and MHC molecules in GBM treatment [47]. Another molecular biomarker of glioma subtypes is the methylated form of O6-methylguanine-DNA methyltransferase (MGMT) promoter, which has prognostic, predictive and clinical applications [48]. MGMT promoter methylation serves as a prognostic and predictive marker in GBM diagnosis, predicting the response to alkylating drugs like temozolomide (TMZ) in glioma patients. Elevated MGMT gene expression levels are associated with treatment resistance, while epigenetic silencing through MGMT gene promoter methylation predicts a positive response to TMZ treatment [49, 50]. The association between MGMT promoter methylation and survival outcomes underscores its potential as a stratification factor for patients with LGG, impacting progression-free survival (PFS) and OS rates [51]. Despite recent advances in the understanding of PBTs and immunity [52], the aim of the present study was to investigate tumor-infiltrating lymphocytes (TILs) and inflammatory markers in patients with different histological types of PBTs in order to correlate the different grade of differentiation and biological and clinical aggressiveness of these neoplasms with the immunological signature.

## Materials and methods

### Patient cohorts, tissue samples and ethics statement

The retrospective study was conducted on 158 brain tumour samples. Tissue samples were collected from Caucasians adult patients with astrocytoma, GBM and meningioma who underwent neurosurgical resection in various local hospitals and were diagnosed at AORN Antonio Cardarelli, Naples, Italy from 2005 to 2015. The histological diagnosis of PBTs was verified by two pathologists (Roberto Monaco and Severo Campione) according to the 2007 World Health Organisation (WHO) classification of CNS tumours [53]. The study was approved by the Local Ethic Committee of the University Hospital of Campania, “Luigi Vanvitelli” Committee, Naples, Prot. N. 30744/i/2022, and the ethical principles defined by the Declaration of Helsinki were followed.

### Tissue microarray (TMA) construction

Formalin-fixed paraffin-embedded (FFPE) representative tissue blocks from 158 patients with PBTs were evaluated and selected with the visual inspection of haematoxylin and eosin-stained slides sections by two pathologists (Roberto Monaco and Severo Campione). Cases with scarce or necrotic material, not representative of the whole tumor, were excluded. Cores were drawn from viable regions of tumour with a 1 mm coring needle using a semi-automated tissue arrayer (Galileo TMA CK 3600, ISENET). Tumour tissue cores were placed in duplicate into 7 paraffin blocks. Microarray blocks were then sectioned in 3–4 µm thickness and subsequently immunostained.

### Immunohistochemistry

Immunohistochemical staining was carried out on FFPE TMA sections to evaluate the expression of CD3, CD4, CD8, CD20, CD138, GzmB, 5-LOX, PD-L1, MGMT,  and TG2. The IHC staining of the slides was performed using the automatic immunostainer BenchMark XT (Ventana, Roche). All markers were stained following the protocol provided by the producer with OptiView DAB IHC Detection Kit (Ventana, Roche). The antibodies and the working conditions are summarized in Table 1.

**Table 1:** List of the antibodies. Technical specifications of the antibodies used for the IHC staining

Marker	Company	Type of antibody	Clone	Dilution (µL)	Buffer pH
CD3	Ventana, Roche, Basel, Switzerland	Rabbit monoclonal	2GV6	Pre-diluted	^a^CC1
CD4	Ventana, Roche, Basel, Switzerland	Rabbit monoclonal	SP35	Pre-diluted	^a^CC1
CD8	Ventana, Roche, Basel, Switzerland	Rabbit monoclonal	SP57	Pre-diluted	^a^CC1
CD20	Ventana, Roche, Basel, Switzerland	Mouse monoclonal	L26	Pre-diluted	^a^CC1
CD138	Cell Marque, California, United States	Mouse monoclonal	B-A38	Pre-diluted	^a^CC1
GzmB	Ventana, Roche, Basel, Switzerland	Rabbit polyclonal	N/A	Pre-diluted	^a^CC1
5-LOX	Cell Signaling Technology, Massachusetts, United States	Rabbit monoclonal	C49G1	Pre-diluted	^a^CC1
PD-L1	Cell Signaling Technology, Massachusetts, United States	Rabbit monoclonal	E1L3N	1/200	^a^CC1
MGMT	Cell Signaling Technology, Massachusetts, United States	Rabbit polyclonal	N/A	Pre-diluted	^a^CC1
TG2	Zedira, Darmstadt, Germany	Mouse monoclonal	XTG17	Pre-diluted	^a^CC1

*aCC1* Cell Conditioning Solution (Ventana Medical Systems, Inc), *N/A* not applicable, *GzmB* granzyme B, *5-LOX* 5-Lipoxygenase, *PD-L1* programmed death-ligand 1, *MGMT* O-6-Methylguanine-DNA Methyltransferase, *TG2* Transglutaminase 2

### TMA slide imaging

All the immunostained slides of TMAs were scanned at 40 × magnification using the Leica Aperio AT2 Scanner. Then, to quantify the expression of biomarkers, the scanned slides were analyzed with the bioimage analysis software Qupath v0.4.3 [54]. All TMA images were independently and blindly reviewed and enumerated by two experienced pathologists (Severo Campione and Marco Montella). In detail, for CD3, CD4, CD8, CD20, CD138 and GzmB, cells were counted in each TMA core and normalized for the number of cells per mm^2^ area. PD-L1 expression was quantified as % of positive tumor cells divided by the total number of viable tumor cells. Instead, for 5-LOX, MGMT, and TG2 the score was calculated by multiplying the intensity of the staining with the percentage of the positive cells showing that staining intensity. The intensity of IHC staining was evaluated as: negative (0), weak (1), moderate (2), strong (3).

### Gene expression, overall survival, and correlation analysis

Gene Expression Profiling Interactive Analysis, version 2 (GEPIA2) tool was used to analyse the potential correlation between the genes encoding for: CD3 (CD3D, CD3E, CD3G), CD4, CD8 (CD8A, CD8B), CD20 (Membrane Spanning 4-Domains A1, MS4A1), CD138 (Syndecan 1, SDC1), GzmB (GZMB), Transglutaminase 2 (TGM2), 5-LOX (Arachidonate 5-Lipoxygenase gene, ALOX5), and MGMT with TCGA dataset sources for GBM and LGG tissues. Spearman’s correlation test was used to analyse the correlation coefficient [55, 56]. Gene expression analysis of the TILs and inflammatory marker genes was generated using GEPIA2 with the following parameters: |Log2FC| Cutoff: 1, p-value Cutoff: 0.01, log scale: log2(TPM + 1) for GBM- and LGG-TCGA and GTEx dataset sources. Overall survival analysis based on the expression status of each gene of interest was obtained using the “Survival Analysis” module of GEPIA2 with default parameters including a 95% confidence interval, “Median” Group cut off and, Hazard Ratio calculated based on the Cox PH model.

### Statistical analysis

The patients were divided into three groups: meningiomas, astrocytomas, and GBMs. Categorical variables were described using absolute and relative frequencies, and group comparisons were performed using the chi-square test. Numerical variables were described using mean and standard deviation (SD) if they exhibited a normal distribution; otherwise, median and interquartile range (IQR) were reported. Group comparisons for numerical variables were conducted using either the one-way test or the Kruskal–Wallis test. In cases where statistically significant differences were found, post-hoc analyses were carried out using pairwise t-tests or pairwise Wilcox tests, with p-values adjusted using the Holm method. When quantitative variables exhibited strong skewness in their distributions, a logarithmic transformation was applied. Correlations between numerical variables were determined using Spearman’s coefficient. Data visualization and analysis were performed with R version 4.3.1.

## Results

### Characteristics of patient cohorts

**Fig. 1:**
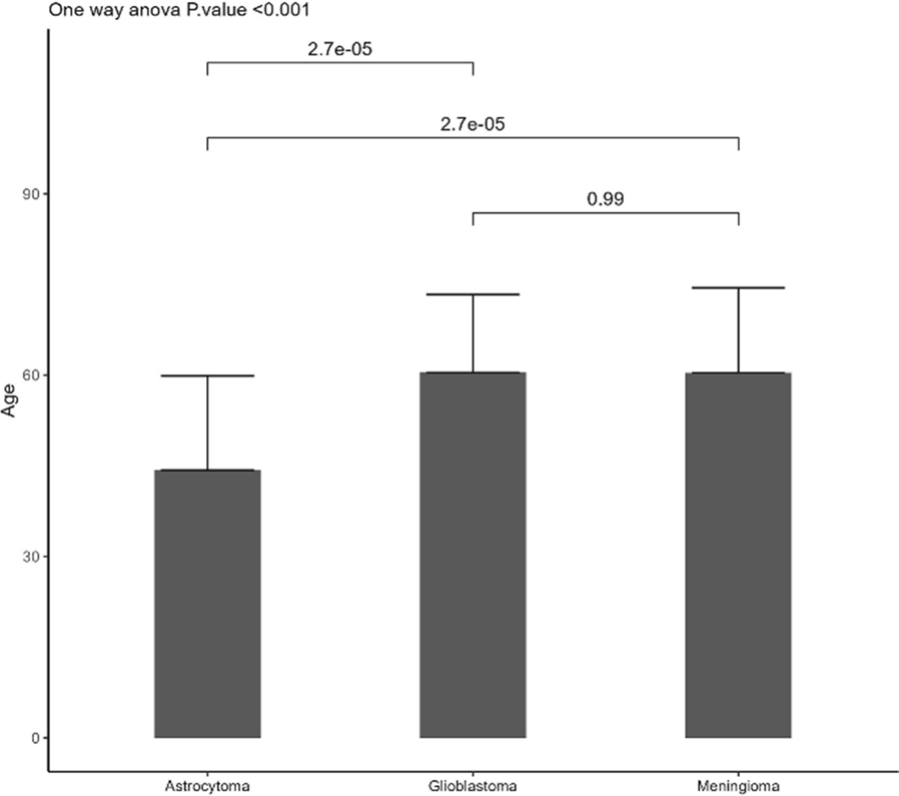
Graphical representation of the correlation between age and PBTs. Average age of individuals diagnosed with meningioma, glioblastoma and astrocytoma expressed as mean ± SD

A total of 158 patients were included in this study. Out of this, 66 (41.7%) meningiomas, 60 (38.0%) GBMs and 32 (20.3%) astrocytomas. Meningioma cohort included 20 (30.3%) males and 46 (69.7%) female patients. GBM cohort included 30 (50.0%) males and 30 (50.0%) females whereas the astrocytoma cohort included 18 (56.2%) males and 14 (43.8%) females. The average age of patients diagnosed with meningioma and GBM was 60.39 and 60.44 years, respectively. No statistical difference was found in the age of diagnosis between meningioma and GBM patients. On the contrary, astrocytoma was diagnosed in younger patients than meningioma and GBM (average age 44.29 years, p < 0.001) (Fig. 1).

IHC staining was performed for all the samples and the results obtained in each cohort are expressed as percentage of positivity, negativity or not detectable for technical reasons (Supplementary Table S1).

### Evaluation of TILs and inflammatory markers in PBT samples by immunohistochemistry

**Fig. 2:**
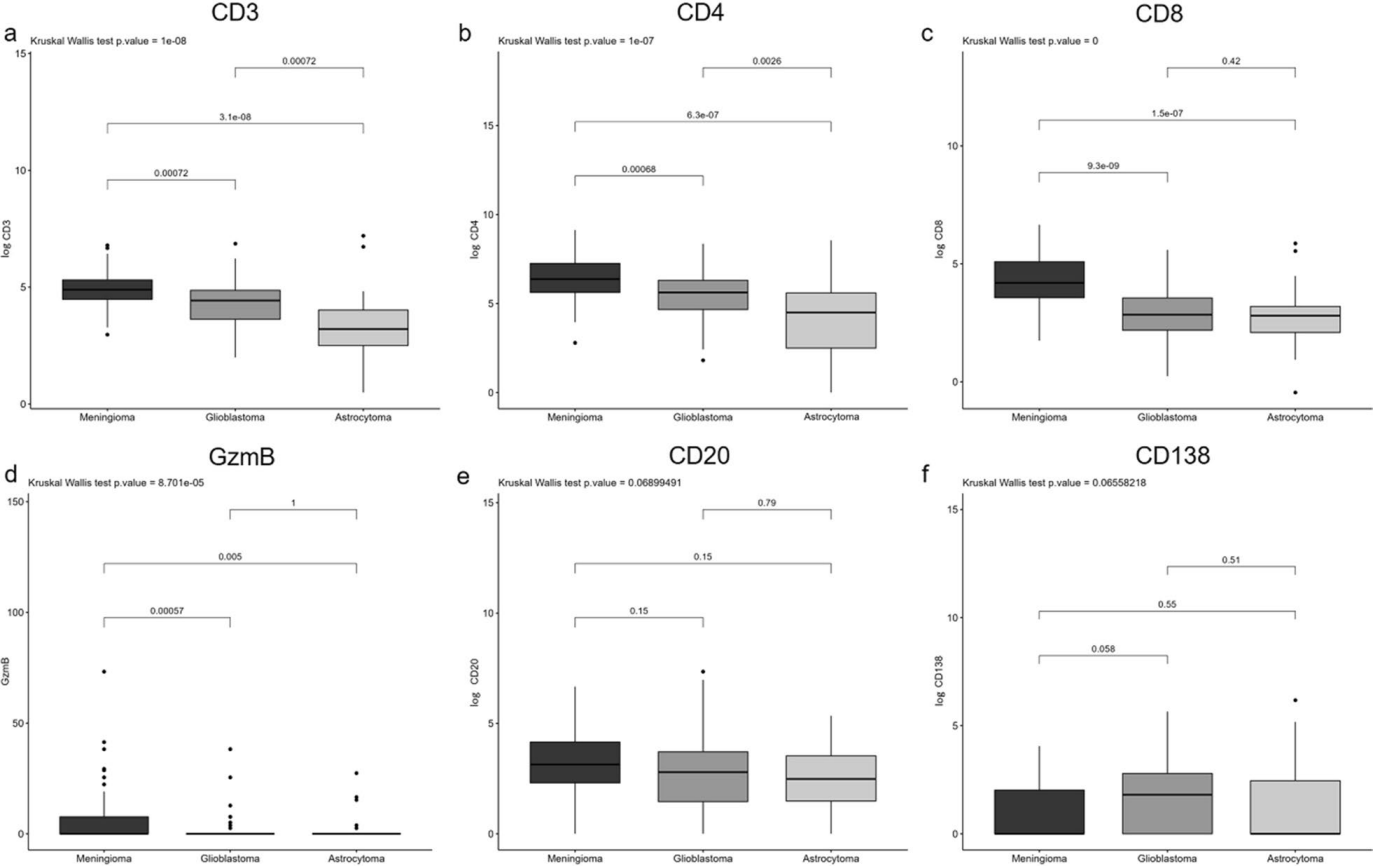
Immunohistochemical evaluation of TILs in PBTs. Box plot diagrams illustrating differences in median of **a** CD3, **b** CD4, **c** CD8, **d** GzmB, **e** CD20 and **f** CD138 expression

**Fig. 3:**
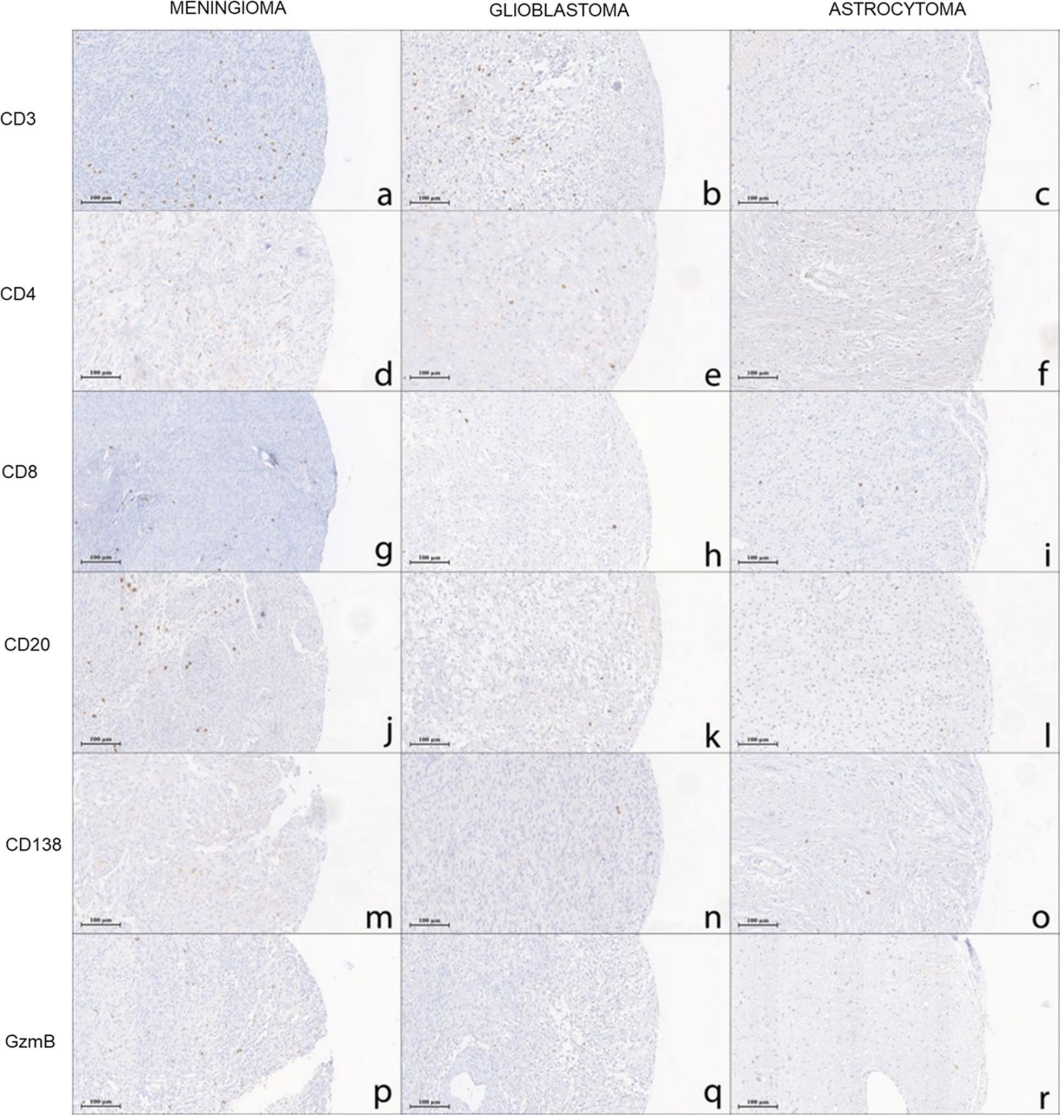
Representative panel of TILs in PTBs. Immunohistochemical images taken at 20X magnification, scale bar 100 µm, for (**a–c**) CD3, (**d–f**) CD4, (**g–i**) CD8, (**j–l**) CD20, (**m–o**) CD138 and (**p–r**) Granzyme B (GzmB) in meningioma, glioblastoma and astrocytoma cohorts. All markers are stained following the protocol provided by the producer with OptiView DAB IHC Detection Kit (Ventana, Roche)

The expression of CD3, CD4 and CD8 in tumour-infiltrating T lymphocytes were localised to the plasma membrane. CD3 and CD8 were positive in all detectable samples (Supplementary Table S1), whereas one astrocytoma sample was negative for CD4 expression. Meningioma samples had a higher number of CD3^+^ TILs (log median 4.89, IQR [4.48, 5.31]) compared to GBM (log median 4.43, IQR [3.63, 4.87]) (p < 0.001) and astrocytoma samples (log median 3.21, IQR [2.50, 4.02]) (p < 0.001). The expression was lower in astrocytoma samples when compared to GBM for CD3^+^ cells (p < 0.001) (Table 2, Figs. 2 and 5). CD4^+^ TILs subset was more enriched in meningiomas (log median 6.37, IQR [5.62, 7.25]) than in glial tumours (p < 0.001), whereas the expression in gliomas was significantly higher in GBMs (log median 5.62, IQR [4.67, 6.30]) than in astrocytomas (log median 4.50, IQR [2.50, 5.60]) (p = 0.0026). Accordingly, CD8^+^ cell population was more present in meningioma (log median 4.19, IQR [3.56, 5.09]) than in glial tumours (p < 0.001). No significant differences in CD8 expression were detected comparing GBM (log median 2.84, IQR [2.19, 3.56]) with astrocytoma (log median 2.80, IQR [2.09, 3.20]). Moreover, CD4^+^ subset was more expressed in all the cohorts than CD8 (Table 2, Figs. 2 and 3). GzmB, CD20, and CD138 were expressed in the cytoplasm of tumour-infiltrating T and B lymphocytes. In line with CD8, the expression of GzmB was significantly higher in the meningioma cohort than in GBMs (p < 0.001) and than in astrocytoma tumours (p < 0.01). On the other hand, no significant results were observed in all cohorts for CD20 and CD138 as illustrated in Figs. 2 and 3.

**Table 2:** Analysis of immunohistochemistry (IHC) scoring of chosen markers in PBTs. Summary of the descriptive analysis and immunohistochemical expression in meningioma, glioblastoma and astrocytoma’s cohorts (N = 158)

	Overall	Meningioma	Glioblastoma	Astrocytoma	p
N	158	66	60	32	
Sex					
M (%)	68 (43.0)	20 (30.3)	30 (50.0)	18 (56.2)	0.020
F (%)	90 (56.9)	46 (69.7)	30 (50.0)	14 (43.8)	
Age (mean (SD))	57.17 (15.32)	60.39 (14.05)	60.44 (12.92)	44.29 (15.60)	< 0.001
log CD3 (mean (SD))	4.49 [3.65, 5.00]	4.89 [4.48, 5.31]	4.43 [3.63, 4.87]	3.21 [2.50, 4.02]	< 0.001
log CD4 (median [IQR])	5.80 [4.77, 6.78]	6.37 [5.62, 7.25]	5.62 [4.67, 6.30]	4.50 [2.50, 5.60]	< 0.001
log CD8 (mean (SD))	3.32 [2.48, 4.33]	4.19 [3.56, 5.09]	2.84 [2.19, 3.56]	2.80 [2.09, 3.20]	< 0.001
GzmB (median [IQR])	0.00 [0.00, 2.55]	0.00 [0.00, 7.64]	0.00 [0.00, 0.00]	0.00 [0.00, 0.00]	< 0.001
log CD20 (median [IQR])	2.83 [1.81, 3.89]	3.14 [2.31, 4.16]	2.79 [1.46, 3.71]	2.48 [1.48, 3.53]	0.069
log CD138 (median [IQR])	0.00 [0.00, 2.42]	0.00 [0.00, 2.02]	1.81 [0.00, 2.79]	0.00 [0.00, 2.46]	0.066
PD-L1 (median [IQR])	0.00 [0.00, 0.00]	0.00 [0.00, 0.00]	0.00 [0.00, 2.50]	0.00 [0.00, 0.00]	0.005
5-LOX TILs score (median [IQR])	0.00 [0.00, 4.17]	0.00 [0.00, 3.38]	0.00 [0.00, 4.38]	1.50 [0.00, 5.08]	0.145
5-LOX cancer cells score (median [IQR])	0.58 [0.00, 2.54]	0.50 [0.00, 4.04]	0.00 [0.00, 1.62]	1.58 [0.33, 2.54]	0.042
MGMT score (median [IQR])	0.21 [0.00, 2.04]	0.25 [0.00, 2.38]	0.33 [0.00, 1.58]	0.00 [0.00, 1.67]	0.558
TG2 score (median [IQR])	1.33 [0.00, 4.00]	0.67 [0.00, 2.00]	1.67 [0.00, 3.33]	2.67 [0.42, 5.17]	0.023
CD3 median (%)					
Negative	79 (50.0)	18 (27.3)	34 (56.7)	27 (84.4)	< 0.001
Positive	76 (48.1)	46 (69.7)	25 (41.7)	5 (15.6)	
Not detectable	3 (1.9)	2 (3.0)	1 (1.7)	0 (0.0)	
CD4 median (%)					
Negative	78 (49.4)	18 (27.3)	37 (61.7)	23 (71.9)	< 0.001
Positive	75 (47.5)	46 (69.7)	22 (36.7)	7 (21.9)	
Not detectable	5 (3.2)	2 (3.0)	1 (1.7)	2 (6.2)	
CD8 median (%)					
Negative	76 (48.1)	12 (18.2)	38 (63.3)	26 (81.2)	< 0.001
Positive	76 (48.1)	51 (77.3)	19 (31.7)	6 (18.8)	
Not detectable	6 (3.8)	3 (4.5)	3 (5.0)	0 (0.0)	
CD20 median (%)					
Negative	76 (48.1)	25 (37.9)	31 (51.7)	20 (62.5)	0.03
Positive	76 (48.1)	39 (59.1)	28 (46.7)	9 (28.1)	
Not detectable	6 (3.8)	2 (3.0)	1 (1.7)	3 (9.4)	
CD138 median (%)					
Negative	1 (0.6)	0 (0.0)	0 (0.0)	1 (3.1)	0.196
Positive	152 (96.2)	64 (97.0)	59 (98.3)	29 (90.6)	
Not detectable	5 (3.2)	2 (3.0)	1 (1.7)	2 (6.2)

**Fig. 4:**
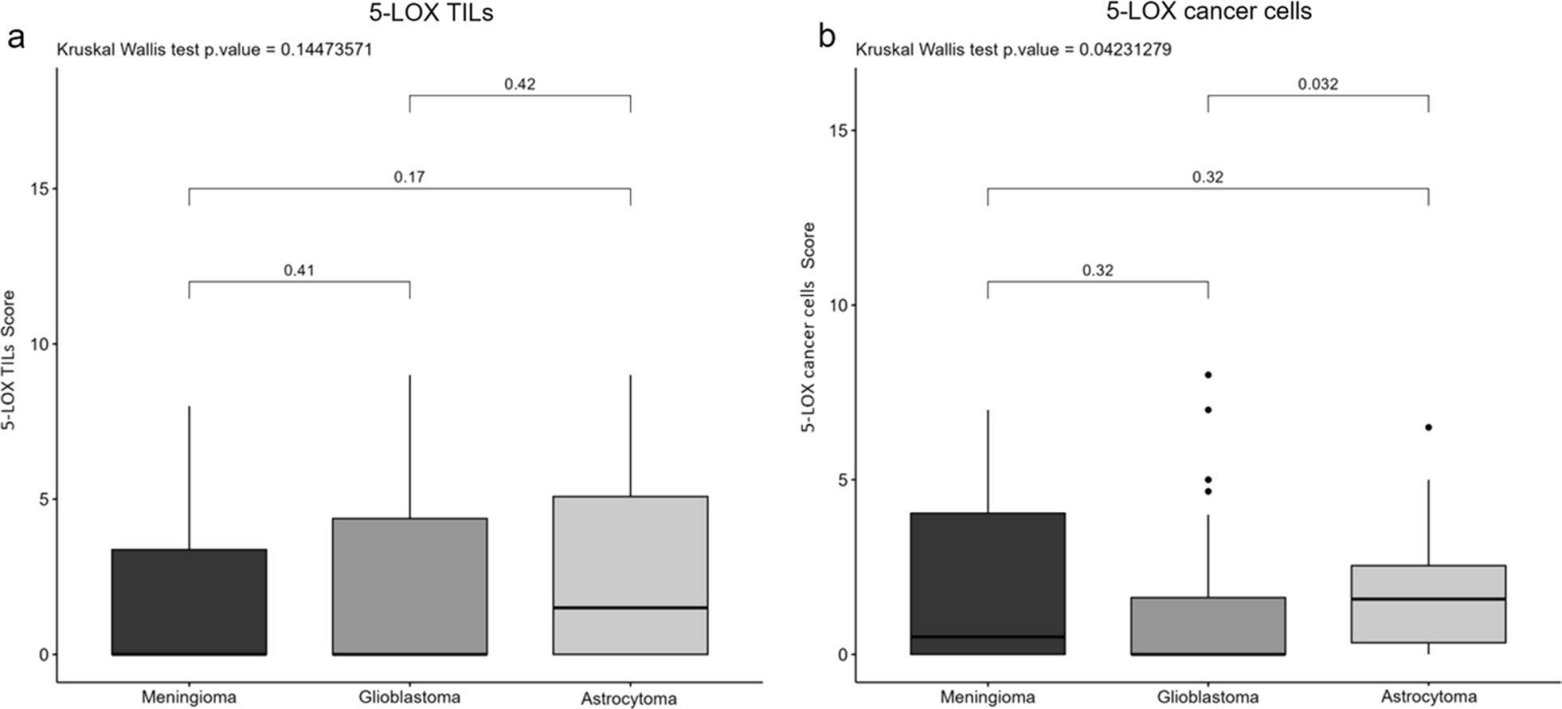
Immunohistochemical evaluation of 5-Lipoxygenase expression (5-LOX) in PBTs. Box plot diagrams illustrating differences in median of **a** 5-LOX TILs and **b** 5-LOX cancer cells expression

**Fig. 5:**
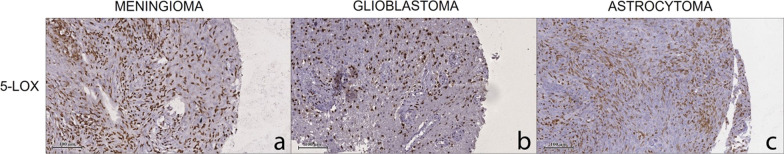
Representative panel of 5-LOX in PTBs. Immunohistochemical images taken at 20X magnification, scale bar 100 µm, for (**a–c**) 5-LOX in meningioma, glioblastoma and astrocytoma cohorts. All markers are stained following the protocol provided by the producer with OptiView DAB IHC Detection Kit (Ventana, Roche)

5-LOX was found expressed in both immune and cancer cells and the signal was localised mostly in the nucleus likely indicating its activation. Out of 33 meningioma samples positive to the immunostaining, only one showed a cytoplasmic expression of 5-LOX in tumour cells (moderate intensity), and two in the immune infiltrate (moderate intensity). In tumour cells, 5-LOX score was significantly higher in astrocytoma samples than in GBMs (p = 0.032). No significant results were detected when the expression of the immune cells was evaluated (Table 2, Figs. 4 and 5).

**Fig. 6:**
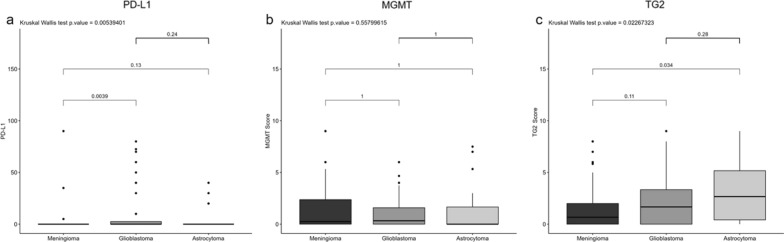
Immunohistochemical evaluation of PD-L1, MGMT, and TG2 in PBTs. Box plot diagrams illustrating differences in median of **a** PD-L1, **b** MGMT, and **c** TG2 expression

**Fig. 7:**
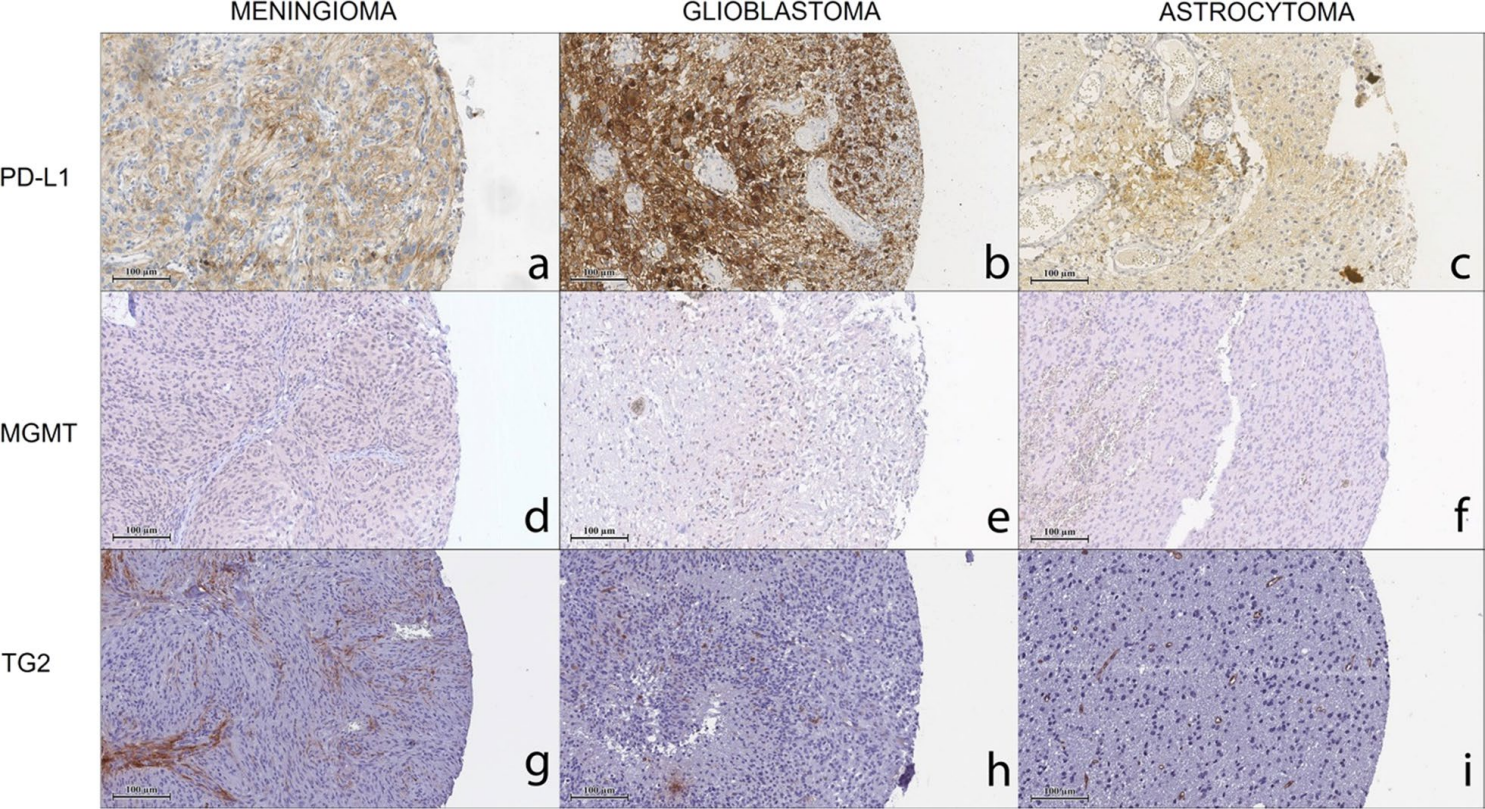
Representative panel of of PD-L1, MGMT, and TG2 in PBTs. Immunohistochemical images taken at 20X magnification, scale bar 100 µm, for (**a**–**c**) PD-L1, (**d**–**f**) MGMT, and (**g**–**i**) TG2 in meningioma, glioblastoma and astrocytoma cohorts. All markers are stained following the protocol provided by the producer with OptiView DAB IHC Detection Kit (Ventana, Roche)

PD-L1 expression was recorded in the cytoplasm of tumour cells, although the rate of PD-L1-positive cases in our cohorts was rather low. Among 158 patients, only 21 (13.3%) showed a positive expression of PD-L1, while 129 (81.6%) were negative and in the remaining 8 (5.1%) not detectable. GBM showed a higher number of positive cases, 13 (21.7%), with the respect to 5 (15.6%) in astrocytomas, and 3 (4.5%) in meningiomas (Supplementary Table S1). No significant differences in PD-L1 expression were detected between the two glial cohorts, neither in meningiomas versus astrocytomas (Table 2, Figs. 6 and 7). Nuclear expression of MGMT did not show any significant differences among the cohorts. On the other hand, TG2 positive cancer cells appeared to be more present in astrocytomas (median 2.67, IQR [0.42, 5.17]) than in meningiomas (median 0.67, IQR [0.00, 2.00]) (p = 0.034). No significant and differential expression was found between glial tumours (Table 2, Figs. 6 and 7).

### Correlation analysis of TILs and inflammatory markers expression in PBTs

**Fig. 8:**
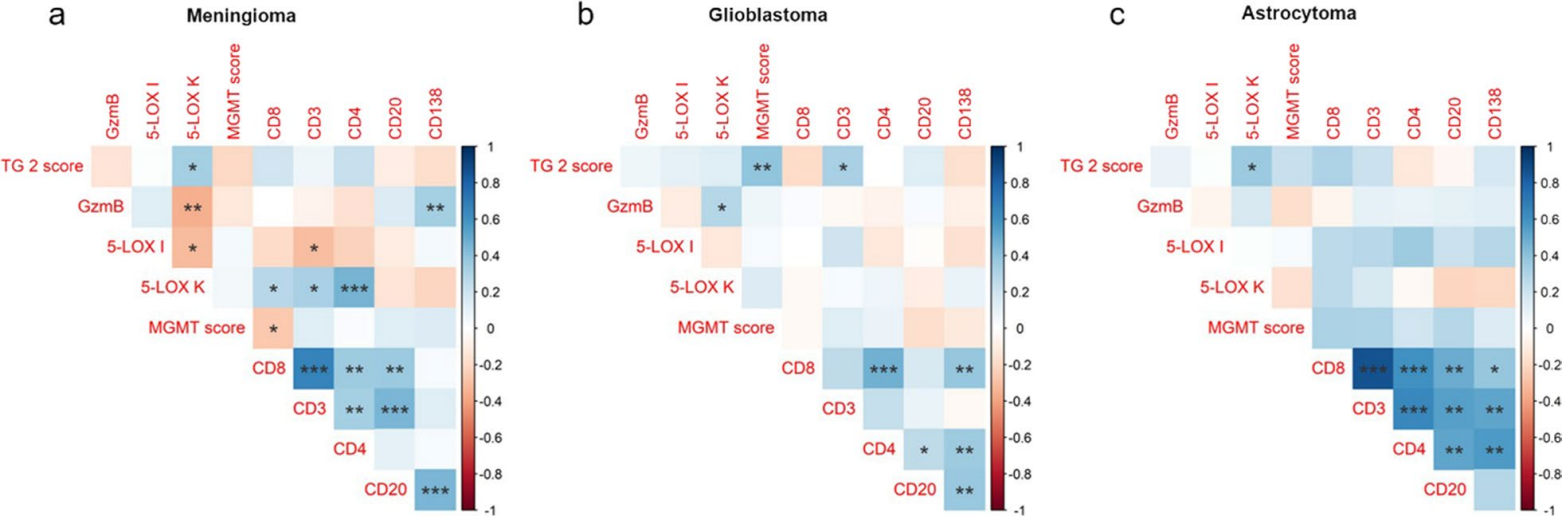
Graphical representation of the interaction between chosen markers in PTBs. Correlation matrix of the immunohistochemical expression of TILs and inflammatory markers in **a** meningioma (N = 66), **b** GBM (N = 60), and **c** astrocytoma (N = 32). The strength of the Spearman’s correlation analysis is represented by the color intensity of each spot, positive in blue and negative in red. *p < 0.05, **p < 0.01, and ***p < 0.001

In order to study the correlation between the tumor immunoregulatory pattern and relevant markers associated with PBTs, a correlation matrix of the screened markers was performed. We have firstly analyzed these correlations in meningiomas. Positive correlations were found between the expression of 5-LOX in tumour cells with TG2 (r = 0.34, p = 0.010), CD3 (r = 0.32, p = 0.013), CD4 (r = 0.47, p < 0.001), and CD8 (r = 0.27, p = 0.039), respectively, whereas 5-LOX in tumour cells negatively correlated to GzmB (r = -0.35, p = 0.006). 5-LOX in immune cells was inversely associated with CD3 (r = − 0.30, p = 0.020) and with the expression of 5-LOX in cancer cells (r = − 0.32, p = 0.013). Furthermore, positive correlations were also present between TILs markers: CD138 with GzmB (r = 0.33, p = 0.008), CD138 with CD20 (r = 0.46, p < 0.001), CD8 with CD3 (r = 0.67, p < 0.001), CD8 with CD4 (r = 0.35, p = 0.005), CD8 with CD20 (r = 0.37, p = 0.004), CD3 with CD4 (r = 0.33, p = 0.008), CD3 with CD20 (r = 0.45, p < 0.001). Finally, MGMT expression was inversely correlated to CD8 (r = − 0.26, p = 0.040) (Fig. 8a). Regarding GBM, the expression of 5-LOX in tumour cells positively correlated only with GzmB (r = 0.29, p = 0.034) (Fig. 8b). Moreover, the Spearman analysis showed a positive correlation for the expression of the TILs markers: CD8 with CD4 (r = 0.47, p < 0.001), CD8 with CD138 (r = 0.39, p = 0.003), CD4 with CD20 (r = 0.26, p = 0.050), CD4 with CD138 (r = 0.35, p = 0.006), CD20 with CD138 (r = 0.38, p = 0.003) (Fig. 8b). In addition, TG2 expression correlated with MGMT (r = 0.040, p = 0.002) and CD3 (r = 0.32, p = 0.015) only in GBM (Fig. 8b). Regarding astrocytoma, in line with meningiomas, the expression of 5-LOX in tumour cells positively correlated to TG2 (r = 0.26, p = 0.046) (Fig. 8c). In addition, the positive correlation was confirmed for: CD8 with CD3 (r = 0.64, p < 0.001), CD8 with CD4 (r = 0.60, p < 0.001), CD8 with CD20 (r = 0.35, p = 0.007), CD3 with CD4 (r = 0.52, p < 0.001), CD3 with CD20 (r = 0.34, p = 0.002), CD4 with CD20 (r = 0.29, p = 0.004), in the same cohort (Fig. 8c). Although CD138 expression weakly and positive correlated with CD8 (r = 0.14, p = 0.036), CD3 (r = 0.079, p = 0.003) and CD4 (r = 0.21, p = 0.001), no significant correlation was found with CD20 (r = 0.35, p = 0.128). No negative correlations were found in both astrocytoma and GBM cohorts (Fig. 8b, c).

**Fig. 9:**
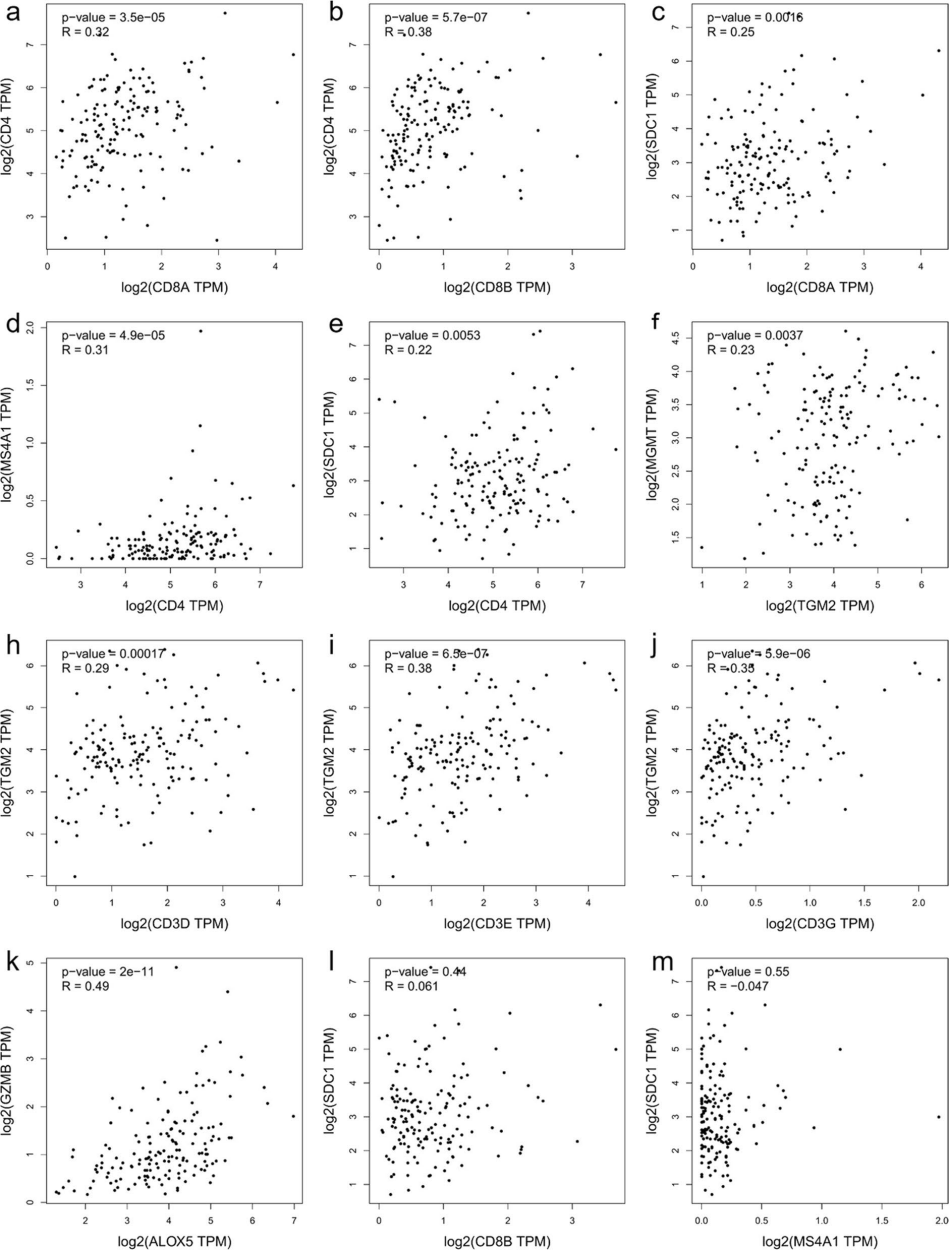
Graphical representation of the interaction between chosen markers in GBM dataset. Scatter plots of the correlation analysis between **a** CD8A with CD4, **b** CD8B with CD4, **c** CD8A with SDC1, **d** CD4 with MS4A1, **e** CD4 with SDC1, **f** TGM2 with MGMT, **h** CD3D with TGM2, **i** CD3E with TGM2, **j** CD3G with TGM2, **k** GZMB with ALOX5, **l** CD8B with SDC1 and **m** SDC1 with MS4A1 are performed on expression data derived from the public cancer portal GEPIA2 using The Cancer Genome Atlas (TCGA)-GBM dataset. Non-log scale is used for calculation and the log-scale axis for visualization. Correlation results are expressed by Spearman’s rank correlation coefficient (R)

Based on the previous results obtained at tissue level, we carried out correlation analyses of the corresponding encoding genes, using TCGA data for GBM and LGG available on GEPIA2. In GBM, significant and weak correlations were confirmed for CD4 with CD8A (r = 0.32, p < 0.001), CD4 with CD8B (r = 0.38, p < 0.001), CD8A with SDC1 (r = 0.25, p = 0.0016), CD4 with MS4A1 (r = 0.31, p < 0.001), CD4 with SDC1 (r = 0.22, p = 0.005), TGM2 with MGMT (r = 0.23, p = 0.004), CD3D with TGM2 (r = 0.29, p < 0.001), CD3E with TGM2 (r = 0.38, p < 0.001), and CD3G with TGM2 (r = 0.35, p < 0.001). Only two studied genes had a moderate and significant positive correlation across GBM samples (GZMB with ALOX5, r = 0.49, p < 0.001) (Fig. 9). The correlation between CD8B and SDC1, and between SDC1 and MS4A1 did not show any significant result.

**Fig. 10:**
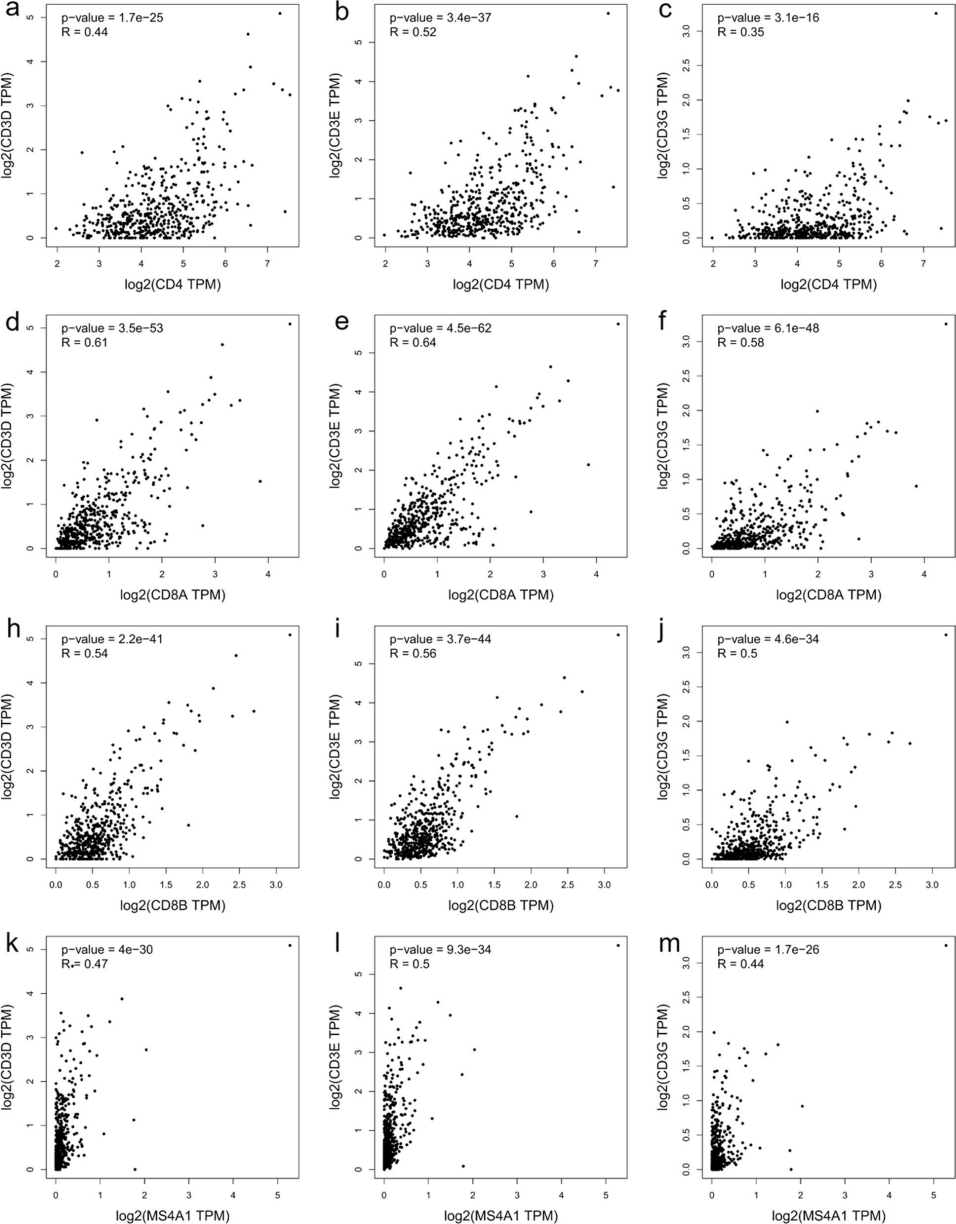
Graphical representation of the interaction between chosen markers in LGG dataset. Scatter plots of the correlation analysis between **a** CD3D with CD4, **b** CD3E with CD4, **c** CD3G with CD4, **d** CD3D with CD8A, **e** CD3E with CD8A, **f** CD3G with CD8A; **h** CD3D with CD8B, **i** CD3E with CD8B, **j** CD3G with CD8B; **k** CD3D with MS4A1, **l** CD3E with MS4A1, **m** CD3G with MS4A1 are performed on expression data derived from the public cancer portal GEPIA2 using The Cancer Genome Atlas (TCGA)-LGG dataset. Non-log scale is used for calculation and the log-scale axis for visualization. Correlation results are expressed by Spearman’s rank correlation coefficient (R)

**Fig. 11:**
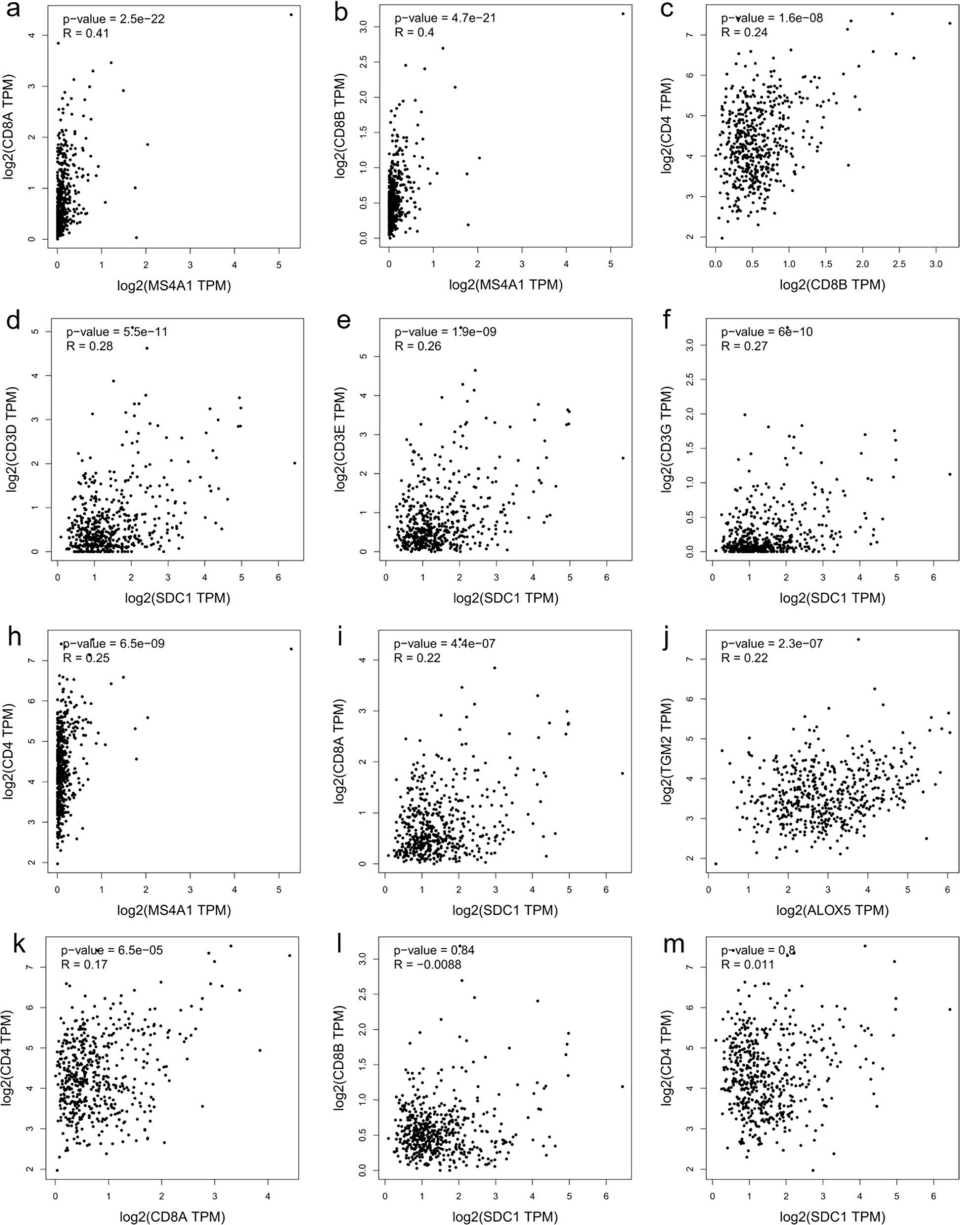
Graphical representation of the interaction between chosen markers in LGG dataset. Scatter plots of the correlation analysis between **a** CD8A with MS4A1, **b** CD8B with MS4A1, **c** CD4 with CD8B, **d** CD3D with SDC1, **e** CD3E with SDC1, **f** CD3G with SDC1, **h** CD4 with MS4A1, **i** CD8A with SDC1, **j** TGM2 with ALOX5, **k** CD4 with CD8A **l** CD8B with SDC1 **m** CD4 with SDC1 are performed on expression data derived from the public cancer portal GEPIA2 using The Cancer Genome Atlas (TCGA)-LGG dataset. Non-log scale is used for calculation and the log-scale axis for visualization. Correlation results are expressed by Spearman’s rank correlation coefficient (R)

On the other hand, in LGG samples, significant moderate correlations were confirmed for CD3D with CD4 (r = 0.44, p < 0.001), CD3E with CD4 (r = 0.52, p < 0.001), CD3D with CD8A (r = 0.61, p < 0.001), CD3E with CD8A (r = 0.64, p < 0.001), CD3G with CD8A (r = 0.58, p < 0.001), CD3D with CD8B (r = 0.54, p < 0.001), CD3E with CD8B (r = 0.56, p < 0.001), CD3G with CD8B (r = 0.50, p < 0.001), CD3D with MS4A1 (r = 0.47, p < 0.001), CD3E with MS4A1 (r = 0.50, p < 0.001), CD3G with MS4A1 (r = 0.44, p < 0.001) (Fig. 10), CD8A with MS4A1 (r = 0.41, p < 0.001), and CD8B with MS4A1 (r = 0.4, p < 0.001) (Fig. 11a, b). Additional evidence of a significant and weak interaction was provided for CD3G with CD4 (r = 0.35, p < 0.001) (Fig. 10), CD4 with CD8B (r = 0.24, p < 0.001), CD3D with SDC1 (r = 0.28, p < 0.001), CD3E with SDC1 (r = 0.26, p < 0.001), CD3G with SDC1 (r = 0.27, p < 0.001), CD4 with MS4A1 (r = 0.25, p < 0.001), CD8A with SDC1 (r = 0.22, p < 0.001), and ALOX5 with TGM2 (r = 0.22, p < 0.001). By contrast, other genes showed a very weak or not significant correlation: CD4 with CD8A (r = 0.17, p < 0.001), CD8B with SDC1 (r = − 0.0088, p = 0.84), and CD4 with SDC1 (r = 0.011, p = 0.8) (Fig. 11). Correlation analyses using GEPIA2 on GBM and LGG datasets have also been performed including the myeloid markers genes such as PTPRC (CD45), CD68, ITGAM (CD11b), CD14 and CD33 in order to evaluate the interactions between lymphocytes and myeloid cells in regulating the inflammation and tumor immunity in the glioma progression to glioblastoma. As shown in the Supplementary Figs. S1–S4, almost all the myeloid markers were associated with CD3, CD4, ALOX5 and TGM2 genes in GBM; whereas only the gene expression of CD14 and CD33 were correlated to GZMB. In the LGG dataset, almost all the markers were correlated to CD3, CD4, PD-L1 and ALOX5.

### Expression profiling of immune infiltration-related and inflammatory genes in GBM and LGG

**Fig. 12:**
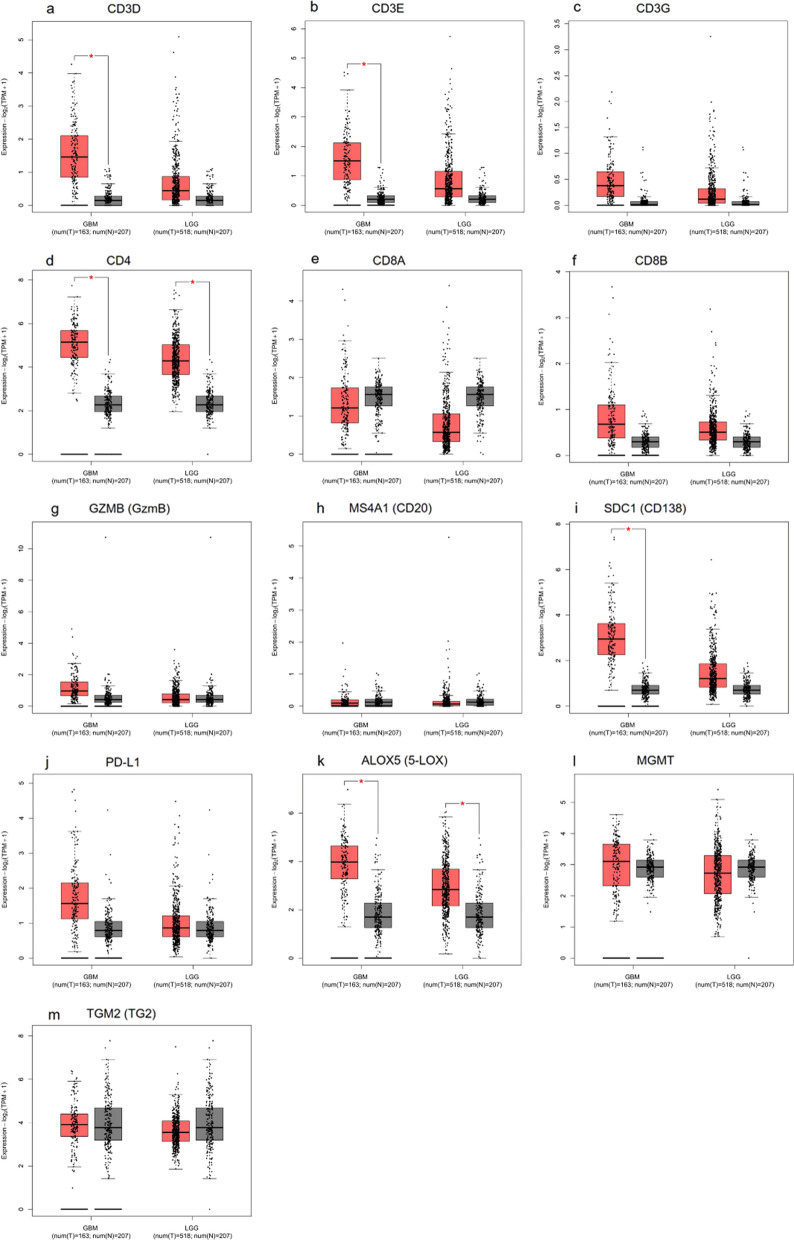
Graphical representation of chosen markers in GBM and LGG dataset. Gene expression analysis on RNA-seq data from TCGA and GTEx samples using GBM and LGG datasets performed with GEPIA2. Gene expression for **a** CD3D, **b** CD3E, **c** CD3G, **d** CD4, **e** CD8A, **f** CD8B, **g** GZMB (GzmB), **h** MS4A1 (CD20), **i** SDC1 (CD138), **j** PD-L1, **k** ALOX5 (5-LOX), **l** MGMT and **m** TGM2 (TG2) is reported as log2(TPM + 1) in tumour samples (GBM: red, T = 163; LGG: red, T = 518) and normal tissue (GBM: grey, N = 207; LGG: grey, N = 207). Significant differences are shown with an asterisk: *p-value ≤ 0.01

To extend the evaluation of immune infiltration-related and inflammatory mechanisms from the protein level to the transcript level, we explored the TCGA and GTEx resources available on GEPIA2 and supported a comparison analysis between GBM and LGG. The expression level of the genes in GBM (Tumour, T = 163) and LGG (Tumour, T = 518) was compared with normal brain samples (Normal, N = 207). The analysis indicated a significant increase in CD3D, CD3E and SDC1 expression in GBM compared to control tissues. CD4 and ALOX5 gene expression level significantly differed between tumour samples when compared to normal brain tissue in both GBM and LGG. However, the expression of CD3D, CD3E and SDC1 was not altered in LGG versus controls indicating their specificity in GBM pathogenesis. No significant differences were found in CD3G, CD8A, CD8B, GZMB, MS4A1, PD-L1, MGMT, and TGM2 (Fig. 12). Gene expression analysis on the myeloid markers for GBM and LGG samples is shown in Figure S5.

### Overall survival analysis based on immune infiltration-related and inflammatory genes in GBM and LGG

**Fig. 13:**
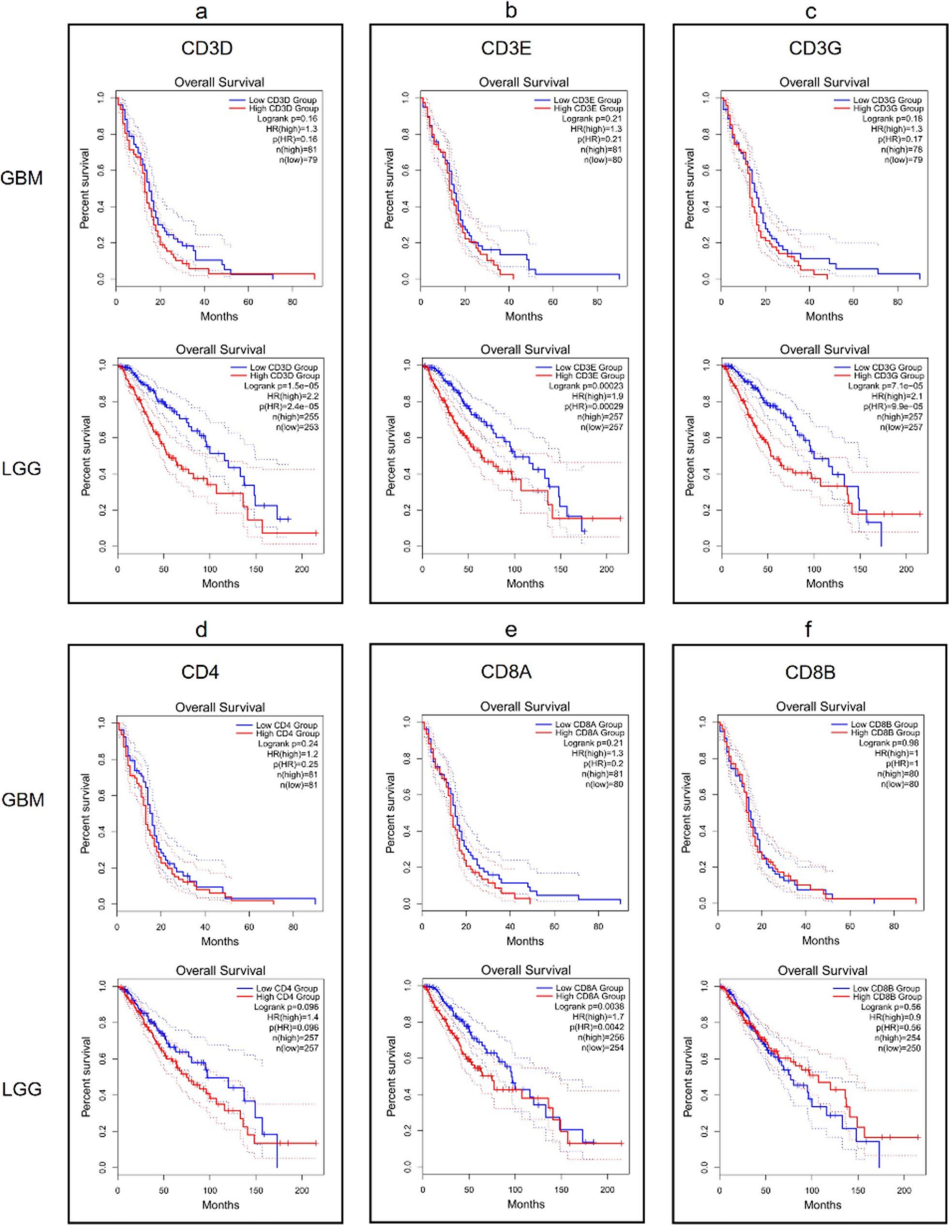

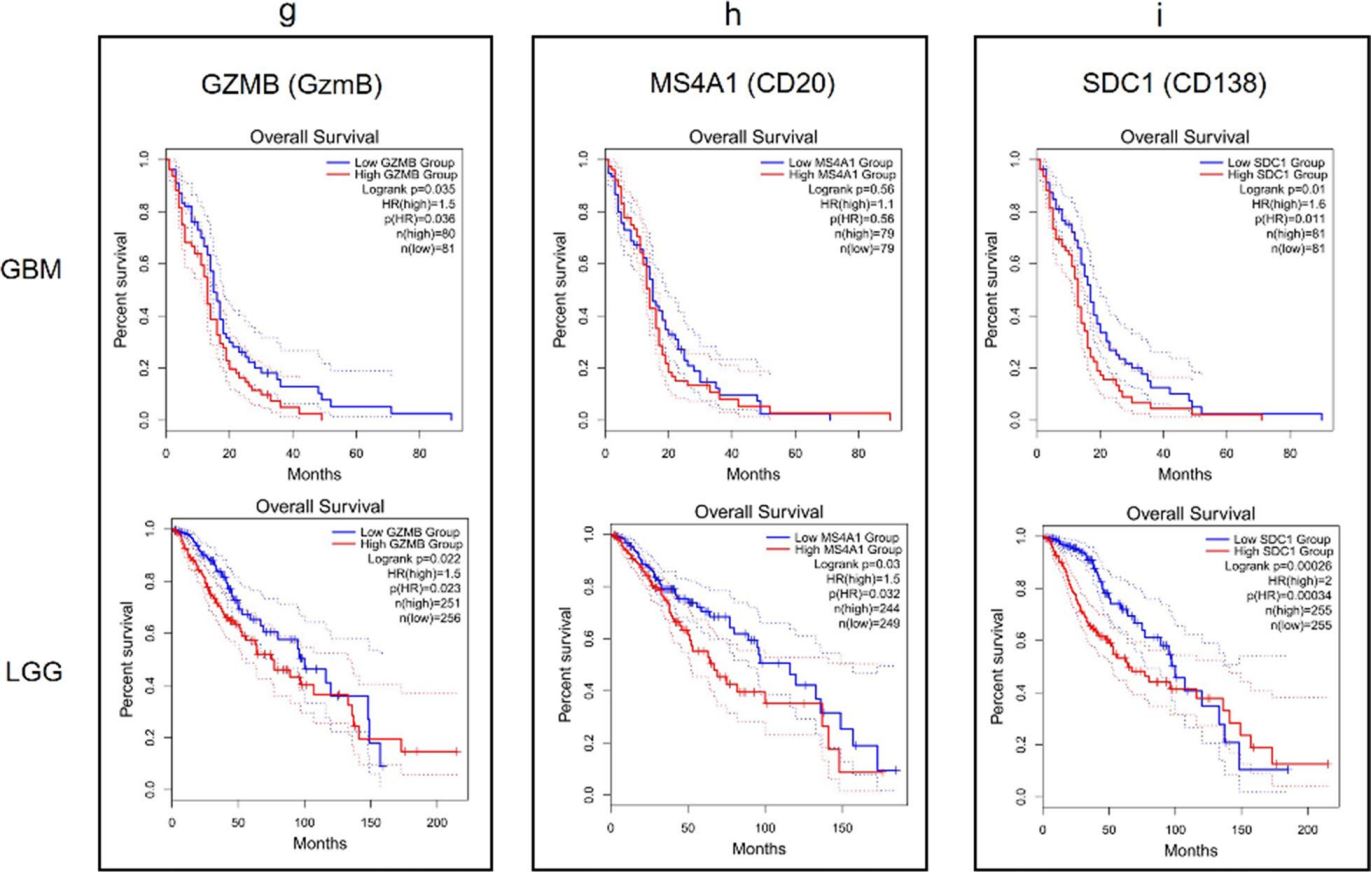
Kaplan–Meier plots for gene expression from the GEPIA2 tool in GBM and LGG cohorts. ‘N’ represents the size of the groups involved in the study with high (red) and low (blue) expression of **a** CD3D; **b** CD3E; **c** CD3G; **d** CD4; **e** CD8A; **f** CD8B; **g** GZMB (GzmB); **h** MS4A1 (CD20); **i** SDC1 (CD138)

**Fig. 14:**
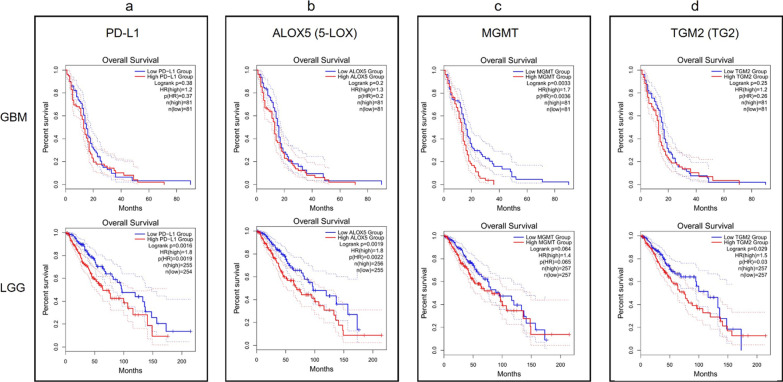
Kaplan–Meier plots for gene expression from the GEPIA2 tool in GBM and LGG cohorts. ‘N’ represents the size of the groups involved in the study with high (red) and low (blue) expression of **a** PD-L1; **b** ALOX5 (5-LOX); **c** MGMT; **d** TGM2 (TG2)

The correlation between gene expression and OS of gliomas was assessed by Kaplan–Meier analysis using public databases available on GEPIA2 portal. The results in GBM patients showed that an increased expression of GZMB (HR = 1.5, logrank p = 0.035), SDC1 (HR = 1.6, logrank p = 0.01) and MGMT (HR = 1.7, logrank p = 0.0033) predicted a poor OS, while the results for CD3D, CD3E, CD3G, CD4, CD8A, CD8B, MS4A1, PD-L1, ALOX5 and TGM2 were not statistically significant. On the other hand, the Kaplan–Meier plots in LGG patients indicated that an increased expression of CD3D (HR = 2.2; logrank p = 1.5e-05), CD3E (HR = 1.9; logrank p = 0.00023), CD3G (HR = 2.1; logrank p = 7.1e-05), CD8A (HR = 1.7; logrank p = 0.0038), GZMB (HR = 1.5; logrank p = 0.022), MS4A1 (HR = 1.5; logrank p = 0.03], SDC1 (HR = 2; logrank p = 0.00026), PD-L1 (HR = 1.8; logrank p = 0.0016), ALOX5 (HR = 1.8; logrank p = 0.0019), and TGM2 HR = 1.5; logrank p = 0.029) were associated with worse OS, whereas no significant correlation between CD4, CD8B, and MGMT gene expression in LGG survival outcomes was found (Figs. 13  and  14).

## Discussion

In this study, we have proposed a comprehensive analysis of a panel of both immune and inflammatory markers in order to investigate the inflammatory microenvironment and the immune response in PBTs. In particular, we have investigated the abundance of antitumor immune cells in TILs evaluating CD3^+^ anti-tumour immune population, CD4^+^ helper and CD8^+^ cytotoxic T cell subsets. In contrast to the CD8^+^ cytotoxic T cells which are the most powerful effectors in the anticancer immune response, the exact mechanism of how CD4^+^ T cells are involved in the anti-tumour immunity is less clear [57–60]. While MHC class I molecules are expressed by the majority of tumour types, most cancer cells lack the expression of MHC class II molecules. However, recent studies show that CD4^+^ T cells can promote and maintain CD8^+^ T cell response through cytokines production or having a cytotoxic role in cancer with the secretion of perforins, granzyme as well as PD-1, TRAIL and FasL. Moreover, CD4^+^T cells can modulate angiogenesis and the inflammatory milieu [61–64]. In this study, we showed that among the PBTs, meningiomas had a higher percentage of CD3^+^, CD4^+^ and CD8^+^ T cells. Previous research has indicated that the density of CD3^+^, CD8^+^ and PD-1^+^ TILs was inversely associated with the WHO grade in meningioma tumour [65]. In fact, in atypical meningioma, a higher density of CD3^+^ and CD8^+^ TILs were associated with better recurrence free survival and TIL density has been considered as a significant prognostic factor [66]. Being intracranial tumours not confined by the BBB, meningiomas are susceptible to peripheral immune cell infiltration and subsequently can be reachable by immunotherapy [67]. The link between meningioma and immune cell infiltration is well established. Many studies reported that meningiomas often express PD-L1, which correlates with higher grade and worse prognosis, and other immune checkpoints, such as TIM-3, NY-ESO-1, PD-1, PD-L2, B7-H3, and CTLA-4 [68–71]. Medici et al. created a T-cell antigen atlas for meningioma through detailed LC–MS/MS profiling of the immunopeptidome and identified new therapeutic targets for immunotherapy approach [72]. Although several studies reported high expression levels of 5-LOX in meningioma cells suggesting a putative role of 5-LOX in meningioma tumorigenesis [21, 73–75], for the first time we have demonstrated a significant correlation between the expression of 5-LOX and presence of the T cell immune infiltration in meningioma tumours. Recent advances in the field have shown that molecular mechanisms of inflammation and immune response are linked, and several findings indicate that 5-LOX is a crucial signal for T cell proliferation and NF-κB, c-jun and PKC pathways activation [76–80]. In a previous study, in mouse peritoneal macrophages it was shown that a product of 5-LOX, the leukotriene B4 (LTB4), together with the arachidonic acid could directly promote the intracellular activation of TG2 [81]. Here, we have observed a positive correlation between the expression of TG2 and 5-LOX in meningioma. Several studies have reported an increased TG2 expression level in meningioma [82, 83]. Huang et al. reported that increased expression of TG2 could predict risk of both tumour recurrence and progression to higher pathological grades [84]. In addition, recent evidence showed that 5-LOX can contribute to a proliferative and pro-survival effect in glioma cells through a modulation of ERKs phosphorylation, Bcl-2/Bax signalling and via β-catenin-dependent pathway [74, 75, 85]. Moreover, a recent publication from Pan et al., showed a positive correlation between 5-LOX expression and tumour immune infiltration in LGG [86]. In line with these findings, we have reported a positive and significant correlation between the tissue expression of 5-LOX and GzmB in GBM samples. This finding was also confirmed with the correlation analysis performed on TCGA-GBM dataset. Differently from other sample sets, in GBM a positive correlation was also found between TG2 and MGMT expression as well as between TG2 and CD3 expression. Nevertheless, in the astrocytoma cohort we found 5-LOX expression to be significantly and positively correlated to TG2. Interestingly, both enzymes modulate the inflammation pathway, probably also with the activation of the NF-κB pathway [22, 87]. Among gliomas, astrocytomas showed a significant reduction in the levels of CD3^+^ and CD4^+^ lymphocytes when compared to GBM. Consistent with our results, Han et al. demonstrated that a poor prognosis was linked to high CD4^+^ TIL levels combined with low CD8^+^ TIL levels [29]. Conversely, we found that 5-LOX expression was higher in astrocytomas than in GBM. Although previously Nathoo et al. demonstrated that 5-LOX is highly expressed in high-grade astrocytomas [88], a comparison with our study cannot be performed considering that we analysed GBM in contrast with astrocytomas, without distinguishing between low- and high- grade astrocytomas. At transcriptional level, gene expression for ALOX5 was increased in both LGG and GBM in comparison to the normal counterpart, as previously already reported [86]. Data from publicly available datasets also showed a significant increase in CD3D, CD3E and SDC1 (CD138) expression in GBM compared to normal tissues, whereas the expression for the same genes was not significantly modulated in LGG when compared to controls indicating that their increased expression could be related to GBM pathogenesis. No significant differences were found in CD3G, CD8A, CD8B, GZMB (GzmB), MS4A1 (CD20), PD-L1, MGMT, and TGM2 (TG2) in both groups. These findings are surprising given the fact that other groups reported that there is a connection between TG2 activity and the molecular events that regulate the mesenchymal transition in GBM cells [26, 89], although we did not discriminate GBM subtypes in our analysis. Correlation analyses on the myeloid markers suggested that tumor-associated myeloid cells can cooperate with both lymphocytes and inflammatory cells to potentially induce chemoresistance, tumor inflammation and tumor-mediated immune evasion mechanisms involved in the glial tumor progression [90]. The correlation between gene expression and OS of gliomas were assessed by Kaplan–Meier analysis using public databases available on GEPIA2 portal. OS analysis confirmed that the expression of GZMB was related to a poor survival in both astrocytoma and GBM, suggesting that mechanisms of survival are activated in PBTs that protect them from the activation of immune effectors. This hypothesis is additionally supported by our findings that show higher PD-L1 expression in GBMs if compared to meningiomas. This difference was also found between GBMs and astrocytomas even if the difference did not reach the statistical significance likely due to the size of the population included in our study. These results confirm the clinical evidence of the immunological resistance of GBMs that could be explained only in part by the existence of the BBB, but that can be based upon the molecular characteristics of the tumour cells that make them more prone to immunological evasion [91]. In fact, recently great emphasis and promises have been shown by the use of immunological checkpoint inhibitors (ICIs) in many cancers but deluding results were achieved in 3 recent trials in GBMs [92–94]. Similar results were also obtained with CAR-T cell therapy through some pilot studies performed on a small number of patients [95, 96]. In this view, we have found an increased expression of the inflammatory marker 5-LOX in PBTs if compared with the normal tissue demonstrating higher inflammation that could induce, in turn, an increased NF-κB activation [97]. In this light, it was demonstrated that NF-κB inhibitors such as bortezomib can induce immunogenic activation in multiple myeloma cells through the induction of apoptosis and release of damage-associated molecular patterns [98, 99]. Even if the findings of this study have to be seen in light of some limitations related to the size of the population included in the study and the use of a previous classification system for CNS tumours (2007 CNS WHO), the use of either proteasome or 5-LOX inhibitors could be thus explored in order to sensitize PBT cells to ICIs.

## Conclusion

We have found a positive and significant correlation between the expression of 5-LOX and GzmB in PBTs, both at RNA and protein levels also suggesting their association with the progression from meningioma to astrocytoma and GBMs. Moreover, increased expression of PD-L1, even if not always statistically significant, was found in GBMs if compared with the other two histological subtypes. This could be at least one of the reasons why PBTs appear to be resistant to immunological-based therapeutic approaches even if the presence of a large immunological infiltrate is generally detected. Additional evaluation is required in order to understand the precise modulation of immune sensitivity of PBTs and discover new immune stimulating targets to define new therapeutic approaches in diseases that are presently orphan of active available drugs.

### Supplementary Information

Supplementary Material 1.

## Data Availability

The datasets generated during and/or analysed during the current study are available from the corresponding author on reasonable request.

## Abbreviations

PBTs: Primary brain tumours
GzmB: Granzyme B
5-LOX: 5-Lipoxygenase
PD-L1: Programmed death-ligand 1
MGMT: O-6-methylguanine-DNA methyltransferase
TG2: Transglutaminase 2
MS4A1: Membrane spanning 4-domains A1
IHC: Immunohistochemistry
GBM: Glioblastoma
LGG: Lower grade glioma
CNS: Central nervous system
BBB: Blood-brain barrier
BCB: Blood-cerebrospinal fluid barrier
TME: Tumor microenvironment
EVs: Extracellular vesicles
NK: Natural killer
HGGs: High-grade gliomas
CTLs: Cytotoxic T lymphocytes
OS: Overall survival
SDC1: Syndecan-1
TMZ: Temozolomide
PFS: Progression-free survival
TILs: Tumor-infiltrating lymphocytes
TMA: Tissue microarray
FFPE: Formalin-fixed paraffin-embedded
GEPIA2: Gene expression profiling interactive analysis, version 2
SD: Standard deviation
IQR: Interquartile range
PTPRC: Protein tyrosine phosphatase receptor type C
ITGAM: Integrin subunit alpha M
ICIs: Immune checkpoint inhibitors
