# Supplementary material for: Immune cell infiltration and inflammatory landscape in primary brain tumours
Source: J Transl Med. 2024 May 30;22:521. doi: 10.1186/s12967-024-05309-1 (PMC11140972; doi:10.1186/s12967-024-05309-1)

**Additional file 1 Table S1.** Immunohistochemistry (IHC) scoring of chosen markers in PBTs. IHC staining results expressed as percentage of positivity, negativity or not detectable for technical reasons in meningioma, glioblastoma and astrocytoma’s cohorts (N=158).

|  | **Overall** | **Meningioma** | **Glioblastoma** | **Astrocytoma** |
| --- | --- | --- | --- | --- |
| **N** | 158 | 66 | 60 | 32 |
| **Age (Not detectable)** | 4 (2.5) | 0 (0.0) | 3 (5.0) | 1 (3.1) |
| **CD3 (%)** |  |  |  |  |
| **Negative** | 0 (0.0) | 0 (0.0) | 0 (0.0) | 0 (0.0) |
| **Positive** | 155 (98.1) | 64 (97.0) | 59 (98.3) | 32 (100) |
| **Not detectable** | 3 (1.9) | 2 (3.0) | 1 (1.7) | 0 (0.0) |
| **CD4 (%)** |  |  |  |  |
| **Negative** | 1 (0.6) | 0 (0.0) | 0 (0.0) | 1 (3.1) |
| **Positive** | 152 (96.2) | 64 (97.0) | 59 (98.3) | 29 (90.6) |
| **Not detectable** | 5 (3.2) | 2 (3.0) | 1 (1.7) | 2 (6.2) |
| **CD8 (%)** |  |  |  |  |
| **Negative** | 0 (0.0) | 0 (0.0) | 0 (0.0) | 0 (0.0) |
| **Positive** | 151 (95.6) | 63 (95.5) | 57 (95.0) | 31 (96.9) |
| **Not detectable** | 7 (4.4) | 3 (4.5) | 3 (5.0) | 1 (3.1) |
| **Granzyme B (GzmB) (%)** |  |  |  |  |
| **Negative** | 109 (69.0) | 33 (50.0) | 49 (81.7) | 27 (84.4) |
| **Positive** | 46 (29.1) | 32 (48.5) | 9 (15.0) | 5 (15.6) |
| **Not detectable** | 3 (1.9) | 1 (1.5) | 2 (3.3) | 0 (0.0) |
| **CD20 (%)** |  |  |  |  |
| **Negative** | 22 (13.9) | 7 (10.6) | 11 (18.3) | 4 (12.5) |
| **Positive** | 130 (82.3) | 57 (86.4) | 48 (80.0) | 25 (78.1) |
| **Not detectable** | 6 (3.8) | 2 (3.0) | 1 (1.7) | 3 (9.4) |
| **CD138 (%)** |  |  |  |  |
| **Negative** | 78 (49.4) | 37 (56.1) | 24 (40.0) | 17 (53.1) |
| **Positive** | 75 (47.5) | 27 (40.9) | 35 (58.3) | 13 (40.6) |
| **Not detectable** | 5 (3.2) | 2 (3.0) | 1 (1.7) | 2 (6.2) |
| **PD-L1 (%)** |  |  |  |  |
| **Negative** | 129 (81.6) | 63 (95.5) | 39 (65.0) | 27 (84.4) |
| **Positive** | 21 (13.3) | 3 (4.5) | 13 (21.7) | 5 (15.6) |
| **Not detectable** | 8 (5.1) | 0 (0.0) | 8 (13.3) | 0 (0.0) |
| **MGMT score (%)** |  |  |  |  |
| **Negative** | 70 (44.3) | 30 (45.5) | 23 (38.3) | 17 (53.1) |
| **Positive** | 82 (51.9) | 34 (51.5) | 35 (58.3) | 13 (40.6) |
| **Not detectable** | 6 (3.8) | 2 (3.0) | 2 (3.3) | 2 (6.2) |
| **5-LOX score (%) TILs** |  |  |  |  |
| **Negative** | 82 (51.9) | 39 (59.1) | 29 (48.3) | 14 (43.8) |
| **Positive** | 66 (41.8) | 21 (31.8) | 27 (45.0) | 18 (56.2) |
| **Not detectable** | 10 (6.3) | 6 (9.1) | 4 (6.7) | 0 (0.0) |
| **5-LOX score (%) cancer cells** |  |  |  |  |
| **Negative** | 64 (40.5) | 27 (40.9) | 31 (51.7) | 6 (18.8) |
| **Positive** | 84 (53.2) | 33 (50.0) | 25 (41.7) | 26 (81.2) |
| **Not detectable** | 10 (6.3) | 6 (9.1) | 4 (6.7) | 0 (0.0) |
| **TG2 score (%)** |  |  |  |  |
| **Negative** | 48 (30.4) | 25 (37.9) | 16 (26.7) | 7 (21.9) |
| **Positive** | 101 (63.9) | 36 (54.5) | 41 (68.3) | 24 (75.0) |
| **Not detectable** | 9 (5.7) | 5 (7.6) | 3 (5.0) | 1 (3.1) |

**Additional file 2 Figure S1** Graphical representation of the interaction between chosen markers in GBM dataset. Scatter plots of the correlation analysis between a) CD3E with PTPRC, b) CD3G with PTPRC, c) CD4 with PTPRC, d) ALOX5 with PTPRC, e) TGM2 with PTPRC, f) CD3E with CD68, h) CD3G with CD68, i) CD4 with CD68, j) ALOX5 with CD68, k) TGM2 with CD68, l) ALOX5 with ITGAM and m) CD4 with ITGAM n) TGM2 with ITGAM are performed on expression data derived from the public cancer portal GEPIA2 using The Cancer Genome Atlas (TGCA)-GBM dataset. Non-log scale is used for calculation and the log-scale axis for visualization. Correlation results are expressed by Spearman's rank correlation coefficient (R).


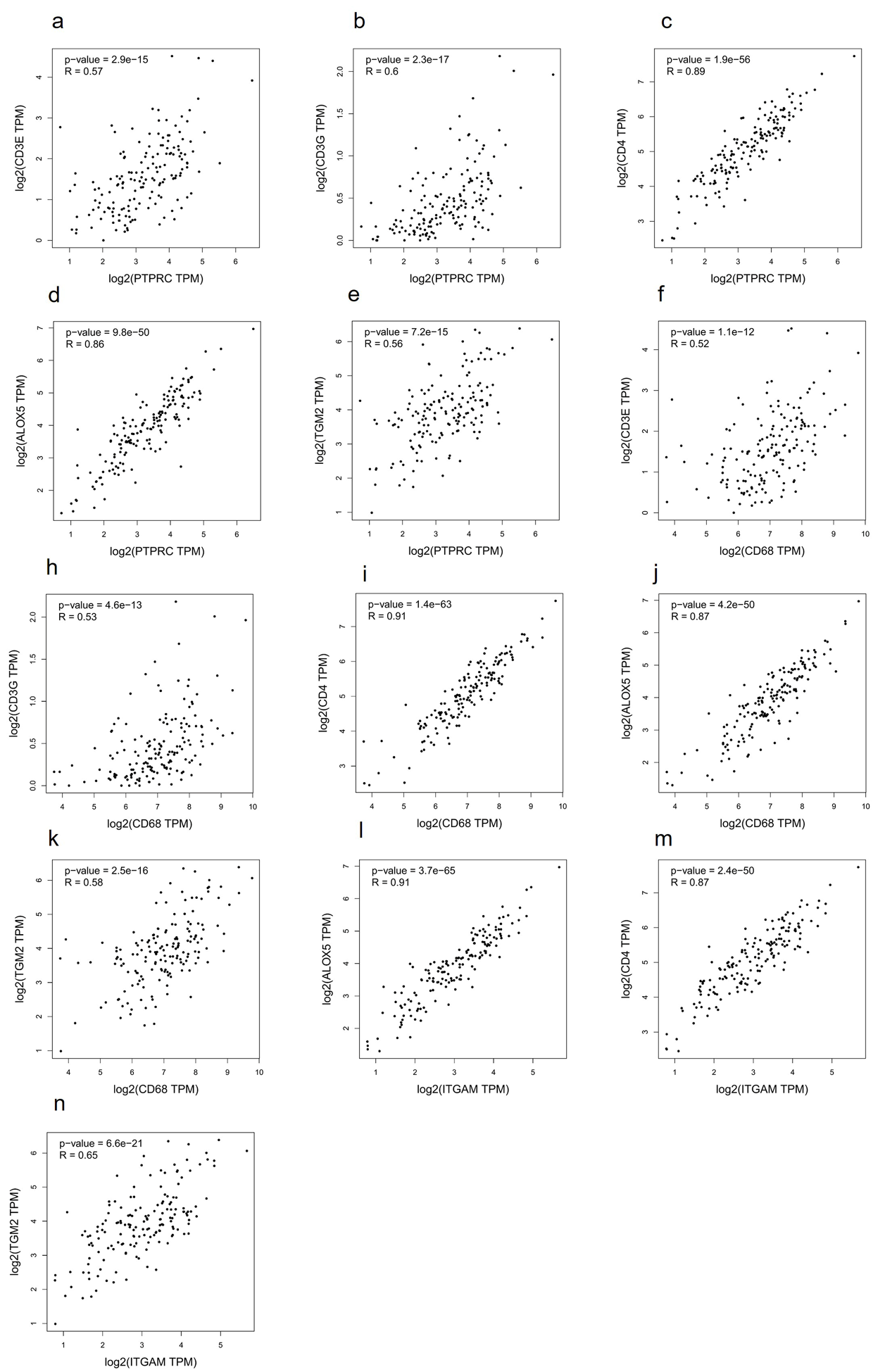


**Additional file 3 Figure S2** Graphical representation of the interaction between chosen markers in GBM dataset. Scatter plots of the correlation analysis between a) CD3D with CD14, b) CD3E with CD14, c) CD4 with CD14, d) GZMB with CD14, e) ALOX5 with CD14, f) TGM2 with CD14, h) CD3D with CD33, i) CD3E with CD33, j) CD3G with CD33, k) CD4 with CD33, l) GZMB with CD33 and m) ALOX5 with CD33 are performed on expression data derived from the public cancer portal GEPIA2 using The Cancer Genome Atlas (TGCA)-GBM dataset. Non-log scale is used for calculation and the log-scale axis for visualization. Correlation results are expressed by Spearman's rank correlation coefficient (R).


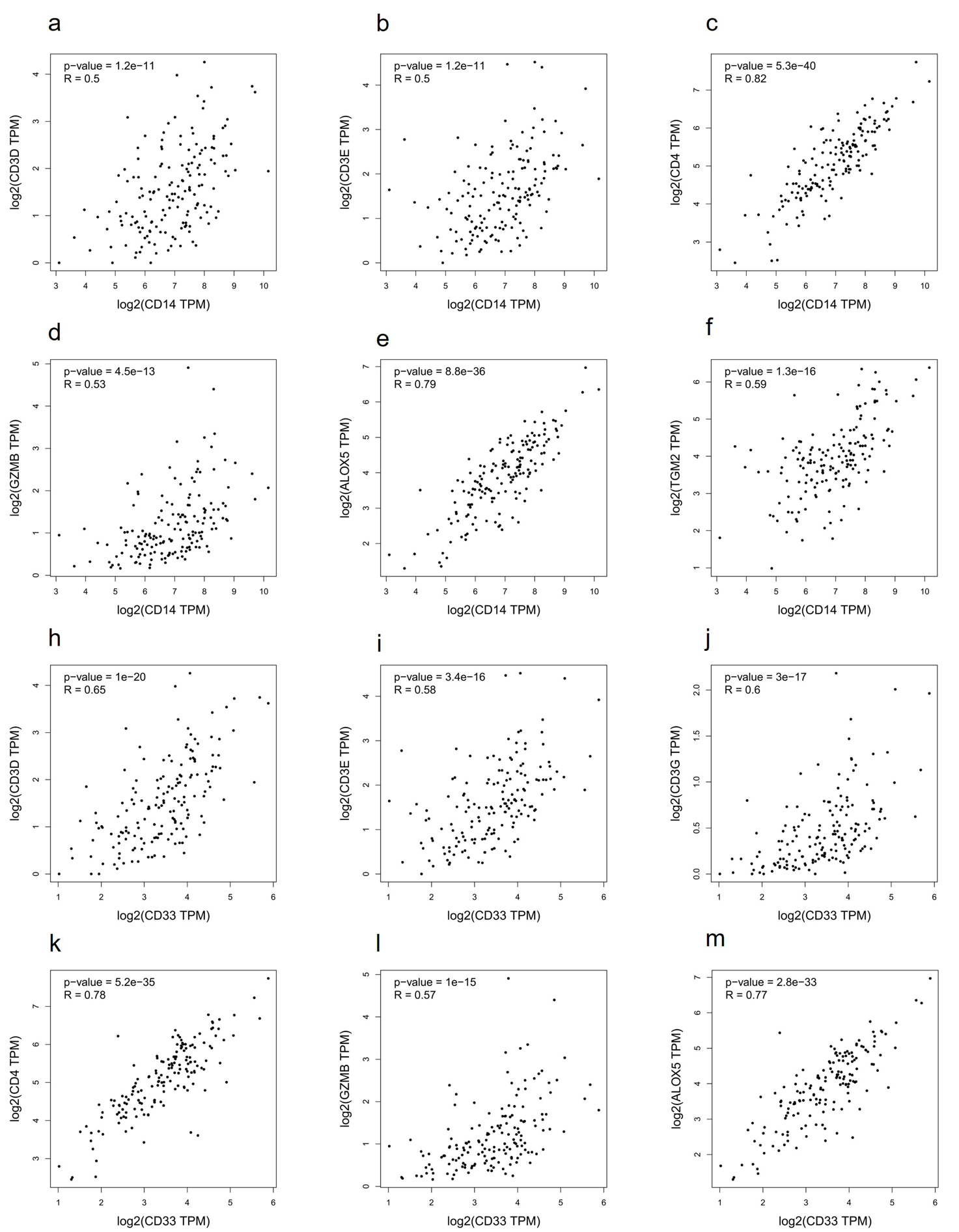


**Additional file 4 Figure S3** Graphical representation of the interaction between chosen markers in LGG dataset. Scatter plots of the correlation analysis between a) CD3E with PTPRC, b) CD4 with PTPRC, c) PD-L1 with PTPRC, d) ALOX5 with PTPRC, e) CD3E with CD68, f) CD4 with CD68; h) PD-L1 with CD68, i) ALOX5 with CD68, j) CD4 with TGAM are performed on expression data derived from the public cancer portal GEPIA2 using The Cancer Genome Atlas (TGCA)-LGG dataset. Non-log scale is used for calculation and the log-scale axis for visualization. Correlation results are expressed by Spearman's rank correlation coefficient (R).


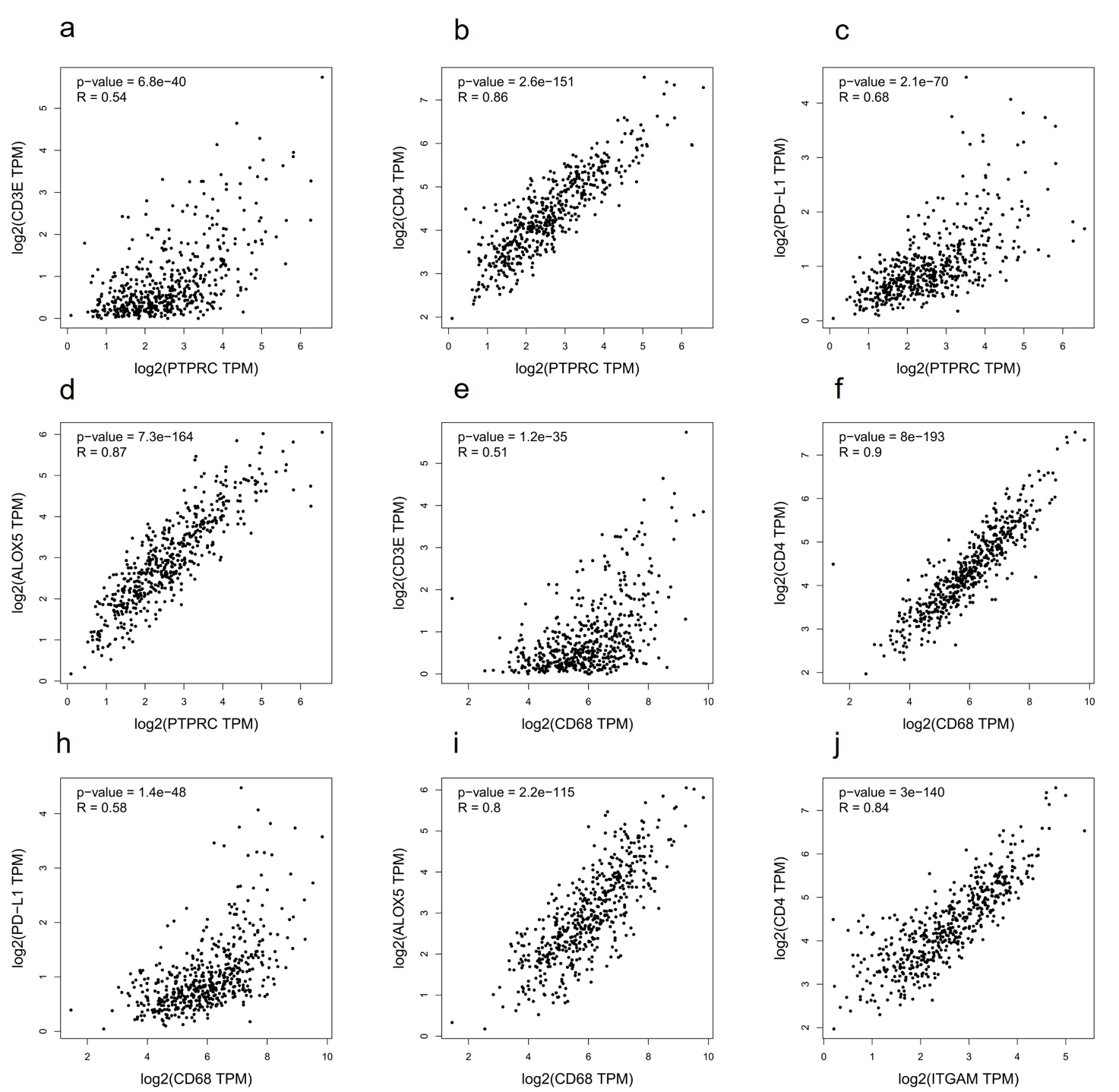


**Additional file 5 Figure S4** Graphical representation of the interaction between chosen markers in LGG dataset. Scatter plots of the correlation analysis between a) PD-L1 with ITGAM, b) ALOX5 with ITGAM, c) ALOX5 with CD14, d) CD4 with CD14, e) PD-L1 with CD33, f) ALOX5 with CD33, h) CD4 with CD33 are performed on expression data derived from the public cancer portal GEPIA2 using The Cancer Genome Atlas (TGCA)-LGG dataset. Non-log scale is used for calculation and the log-scale axis for visualization. Correlation results are expressed by Spearman's rank correlation coefficient (R).


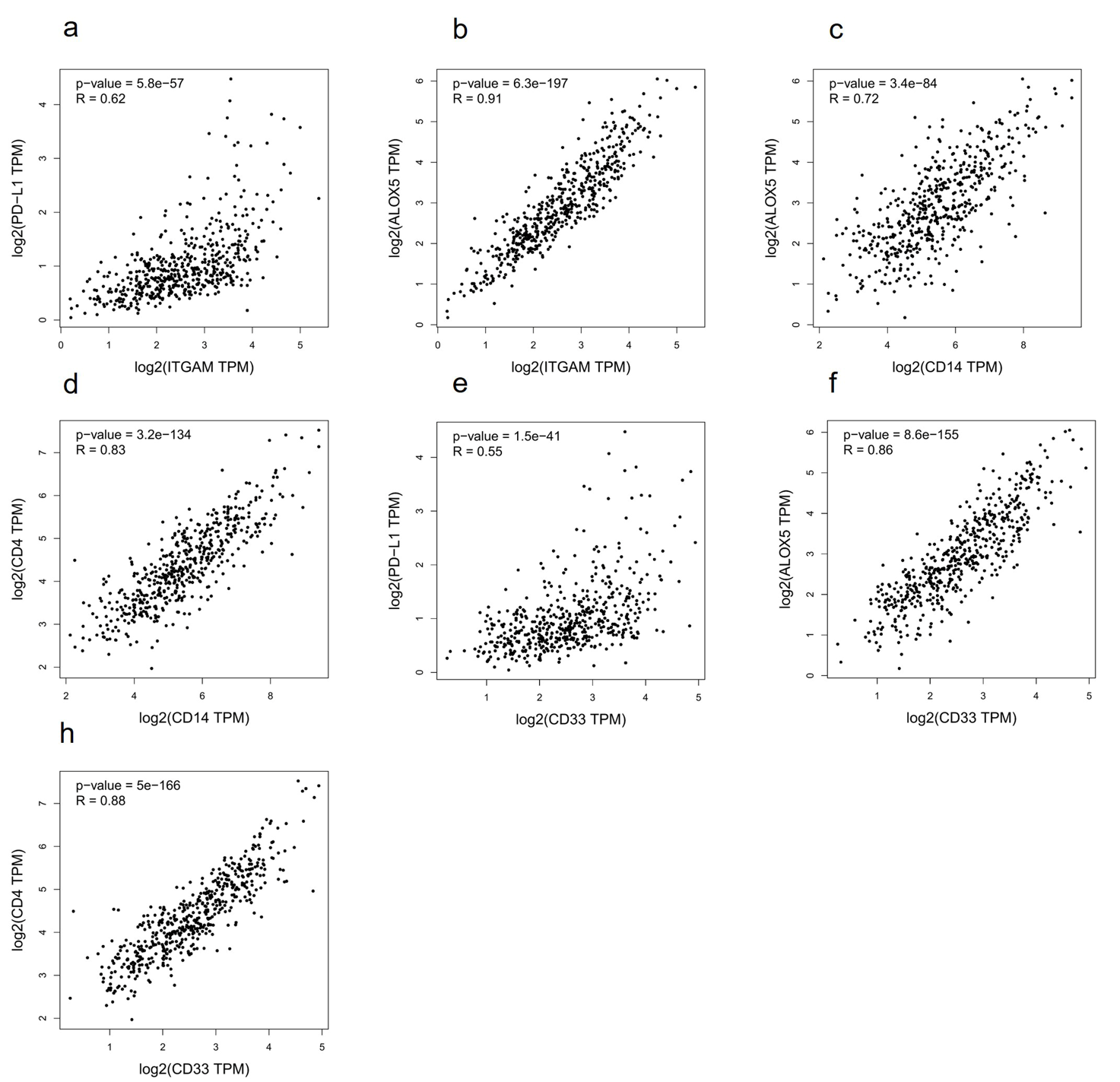


**Additional file 6 Figure S5** Graphical representation of chosen markers in GBM and LGG dataset. Gene expression analysis on RNA-seq data from TCGA and GTEx samples using GBM and LGG datasets performed with GEPIA2. Gene expression for a) PTPRC (CD45), b) CD68, c) ITGAM (CD11b), d) CD14, e) CD33 is reported as log2(TPM + 1) in tumour samples (GBM: red, T = 163; LGG: red, T = 518) and normal tissue (GBM: grey, N = 207; LGG: grey, N = 207). Significant differences are shown with an asterisk: * p-value ≤ 0.01


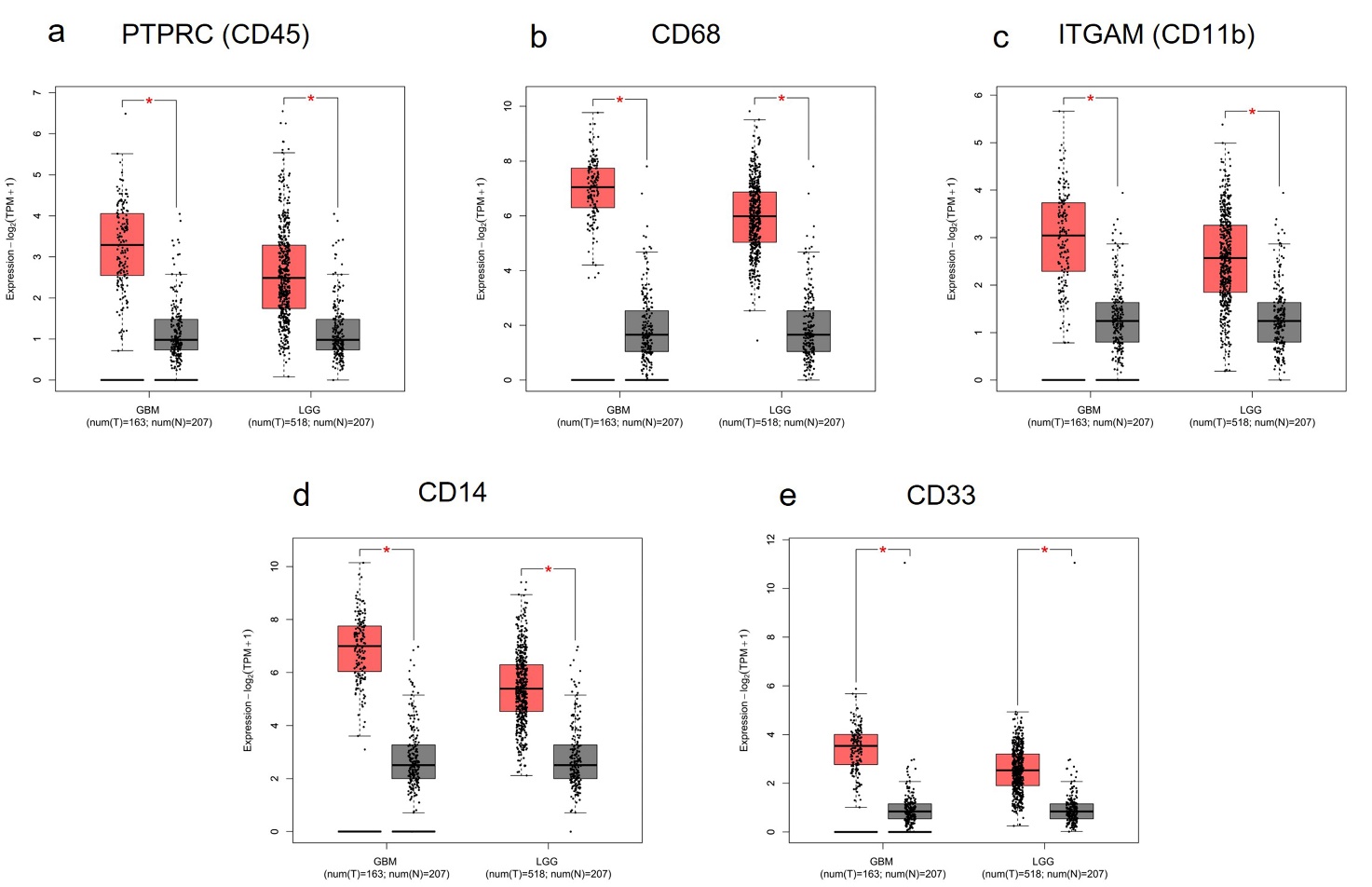

Supplement: Supplementary file 1 — Supplementary Material 1. [file 12967_2024_5309_MOESM1_ESM.docx]
